# Synthesis of sp^2^-Iminosugar Selenoglycolipids as Multitarget Drug Candidates with Antiproliferative, Leishmanicidal and Anti-Inflammatory Properties

**DOI:** 10.3390/molecules26247501

**Published:** 2021-12-11

**Authors:** Elena M. Sánchez-Fernández, Raquel García-Hernández, Francisco Gamarro, Ana I. Arroba, Manuel Aguilar-Diosdado, José M. Padrón, José M. García Fernández, Carmen Ortiz Mellet

**Affiliations:** 1 Department of Organic Chemistry, Faculty of Chemistry, University of Seville, C/Profesor García González 1, 41012 Seville, Spain; mellet@us.es; 2 Instituto de Parasitología y Biomedicina “López-Neyra”, Parque Tecnológico de Ciencias de la Salud, 18016 Granada, Spain; raquelg@ipb.csic.es (R.G.-H.); gamarro@ipb.csic.es (F.G.); 3 Research Unit, Biomedical Research and Innovation Institute of Cádiz, Puerta del Mar University Hospital, Av/Ana de Viya 21, 11009 Cádiz, Spain; anaarroba@gmail.com (A.I.A.); manuel.diosdado@uca.es (M.A.-D.); 4 BioLab, Instituto Universitario de Bio-Orgánica Antonio González, Universidad de La Laguna, C/Astrofísico Francisco Sánchez 2, 38206 La Laguna, Spain; jmpadron@ull.es; 5 Instituto de Investigaciones Químicas, CSIC-University of Seville, Américo Vespucio 49, 41092 Sevilla, Spain; jogarcia@iiq.csic.es

**Keywords:** seleno-sp^2^-iminoglycolipids, multitarget, immunomodulation, cancer, *Leishmania*, inflammation

## Abstract

sp^2^-Iminosugar glycolipids (sp^2^-IGLs) represent a consolidated family of glycoconjugate mimetics encompassing a monosaccharide-like glycone moiety with a pseudoamide-type nitrogen replacing the endocyclic oxygen atom of carbohydrates and an axially-oriented lipid chain anchored at the pseudoanomeric position. The combination of these structural features makes them promising candidates for the treatment of a variety of conditions, spanning from cancer and inflammatory disorders to parasite infections. The exacerbated anomeric effect associated to the putative sp^2^-hybridized N-atom imparts chemical and enzymatic stability to sp^2^-IGLs and warrants total α-anomeric stereoselectivity in the key glycoconjugation step. A variety of *O*-, *N*-, *C*- and *S*-pseudoglycosides, differing in glycone configurational patterns and lipid nature, have been previously prepared and evaluated. Here we expand the chemical space of sp^2^-IGLs by reporting the synthesis of α-d-gluco-configured analogs with a bicyclic (5*N*,6*O*-oxomethylidene)nojirimycin (ONJ) core incorporating selenium at the glycosidic position. Structure–activity relationship studies in three different scenarios, namely cancer, Leishmaniasis and inflammation, convey that the therapeutic potential of the sp^2^-IGLs is highly dependent, not only on the length of the lipid chain (linear aliphatic C_12_ vs. C_8_), but also on the nature of the glycosidic atom (nitrogen vs. sulfur vs. selenium). The ensemble of results highlights the α-dodecylseleno-ONJ-glycoside as a promising multitarget drug candidate.

## 1. Introduction

**Figure 1:**
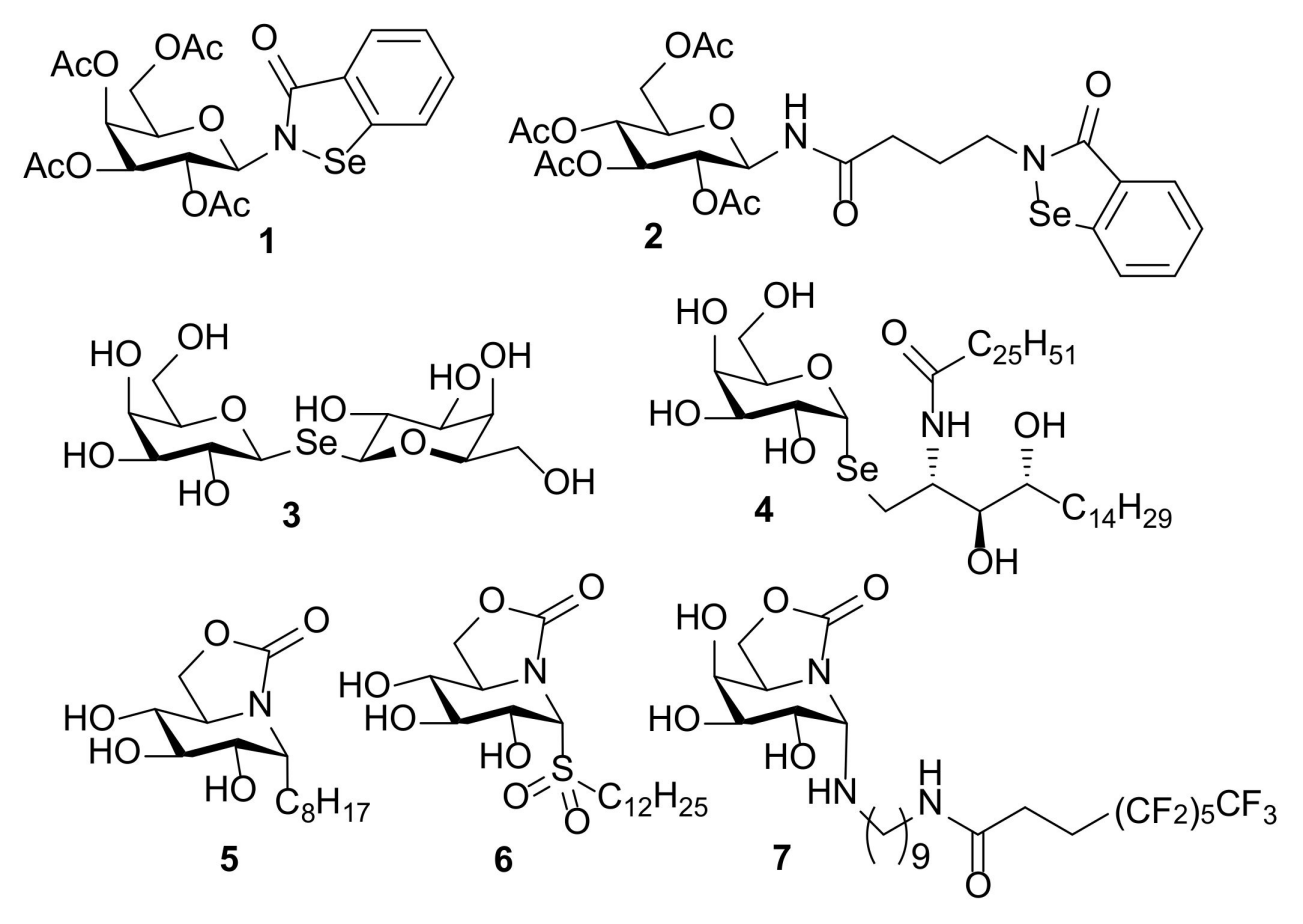
Chemical structures of biologically relevant sugar-based molecules incorporating nitrogen, carbon, sulfur and selenium as glycosidic linkages (**1**–**7**).

The outstanding beneficial effects of selenium-containing molecules in a broad spectrum of pathologies have boosted research in this area over the last two decades [1,2,3,4]. Thus, a large variety of organoselenium compounds encompassing selenide, diselenide, selenocyanate, selenoester, selenocarbamate, selenazo or selenourea derivatives have shown remarkable antitumor [5,6], antiinfective [7], anti-inflammatory [8] and antioxidant [9] properties. In the field of carbohydrates, several selenium-containing products with interesting biological properties have been reported. Some representative examples are depicted in Figure 1. The antimetastatic *N*-glycoside **1** and its analogue **2**, both incorporating the known Ebselen heterocycle in their structures [10], target multiple protein kinase-signalling cascades involved in cancer progression and inflammation [11,12,13,14]. The symmetric selenodigalactoside **3** behaves as a relevant ligand for human galectins [15], whereas α-*Se*-galactosyl ceramide **4** [16], a mimetic of the powerful immunostimulant glycolipid α-galactosyl ceramide (KRN7000) [17], shows interest as a potential adjuvant. In general terms, substitution of the glycosidic oxygen atom by Se results in conjugates that are more robust against enzymatic and chemical degradation, as is also the case with other heteroatoms, such as N or S, empowering the synthesis and biological evaluation of valuable glycomimetics [18,19,20,21,22].

The majority of available methods for the synthesis of *X*-glycosides (*X* = heteroatom other than oxygen) mainly afford the β-anomer or α,β-anomeric mixtures [23,24,25,26], the stereoselective synthesis of α-anomers being consistently more demanding [27,28,29]. sp^2^-Iminosugars remain a unique exception to the rule [30,31,32,33]. The distinctive architecture of these sugar analogues, which bear a pseudoamide-type nitrogen, with substantial sp^2^-hybridation in place of the endocyclic oxygen of monosaccharides, provokes a strong reinforcement of the anomeric effect that compels the axial orientation of pseudoanomeric substituents. This property warrants total α-stereochemical control in glycosylation reactions and imparts chemical and enzymatic stability to the resulting α-pseudoglycosides [34,35], a scenario sharply different to that encountered with classical iminosugars. It has been already capitalized in the design of specific glycosidase inhibitors and effectors [36,37], lectin ligands [38,39,40,41] and tumor-associated carbohydrate antigen (TACA) mimics [42,43], including a sp^2^-iminosugar glycopeptide-based anticancer vaccine [44]. Conjugates conjoining a sp^2^-minosugar glycone and an α-oriented lipid aglycone, generically termed sp^2^-iminosugar glycolipids (sp^2^-IGLs), have also shown promising abilities as innate immune system regulators with anti-inflammatory [45,46,47], anticancer [48,49] and antiparasitic [50,51] behaviors. A variety of sp^2^-IGLs with *C*-, *N*-, *O*- and *S*-pseudoglycosidic bonds are already on record [52,53,54]. Structure–activity relationship studies have shown that (i) the lipid aglycone plays an essential role in the therapeutic effect of the sp^2^-IGLs; and (ii) the nature of the glycosidic functionality bridging the aglycone and lipid moieties is also critical for activity. The configurational profile of the glycomimetic component is comparatively less influencing. For instance, epimeric compounds displaying hydroxylation patterns of stereochemical complementarity with d-glucopyranose or d-galactopyranose behave similarly in different settings [50,53]. Investigations into the molecular mechanism at play revealed that the sp^2^-IGLs interfere with kinase signaling pathways. Thus, in breast cancer cells, the *C*-octyl nojirimycin-related derivative **5** (Figure 1) promoted a reduction in the phosphorylation levels of focal adhesion kinase (FAK) and extracellular signal-regulated kinase 1/2 (ERK1/2) [48], a member of the mitogen-activated protein kinase (MAPK) group. The sulfone derivative **6** and the *N*-glycoside **7** (Figure 1) triggered activation of p38α-MAPK, a master regulator of inflammation. Indeed, computational experiments suggested that sp^2^-IGLs can bind to the lipid-binding pocket of p38 and induce its autophosphorylation [50,55].

**Figure 2:**
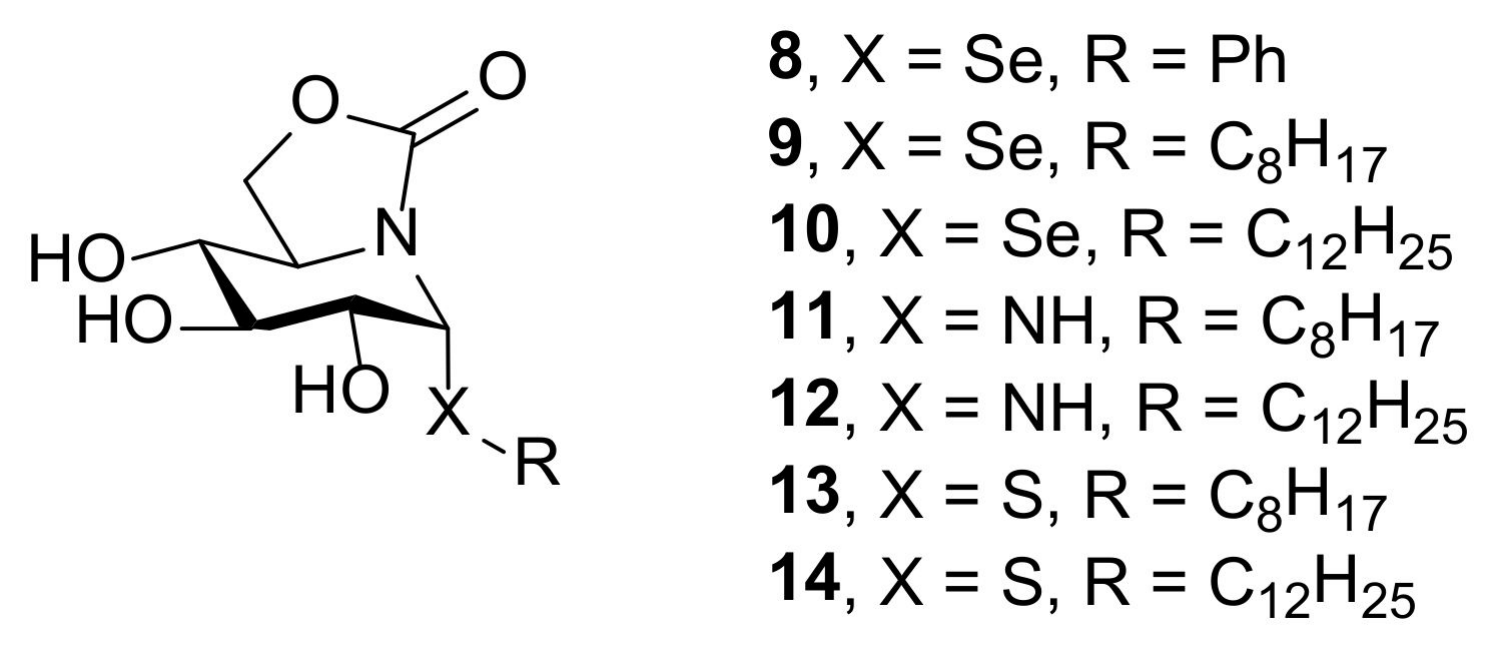
Chemical structures of the sp^2^-IGLs derived from nojirimycin evaluated in this study (**8**–**14**).

Given the therapeutic potential of selenium-containing compounds, the synthesis of seleno-sp^2^-IGLs seemed very appealing in this context. Here we present the stereoselective preparation of the first members of this category, namely compounds **8**–**10** (Figure 2). Their in vitro antiproliferative, leishmanicidal and anti-inflammatory activities, in comparison with *N*-(**11**, **12**) and *S*-(**13**, **14**) analogues, are also reported.

## 2. Results and Discussion

### 2.1. Design and Synthesis

The ensemble of data collected on the immunomodulatory behavior of sp^2^-IGLs [45,46,47,48,49,50,51,52,53,54,55] instils that linear aliphatic tails with a length equal or higher than C_8_ are required to elicit significant anticancer, antiparasitic and/or anti-inflammatory responses. Accordingly, we focused on n-octyl and n-docecyl aglycone lipid tails in this study. As the glycone moiety, the bicyclic 5*N*,6*O*-oxamethylidenenojirimycin (ONJ) core was chosen since ONJ-based conjugates have already demonstrated high promise as drug candidates.

In order to evaluate the impact of the nature of the glycosidic atom on the pharmacological properties, we settled on synthesizing the new seleno-sp^2^-IGLs **9** and **10**, and the homologous amino- and thio-glycosides **11**, **12** and **13**, **14**, respectively. The preparation of the phenyl selenoglycoside **8**, expectedly a negative control, was additionally undertook to test the robustness of the selenoglycosylation reaction.

**Scheme 1:**
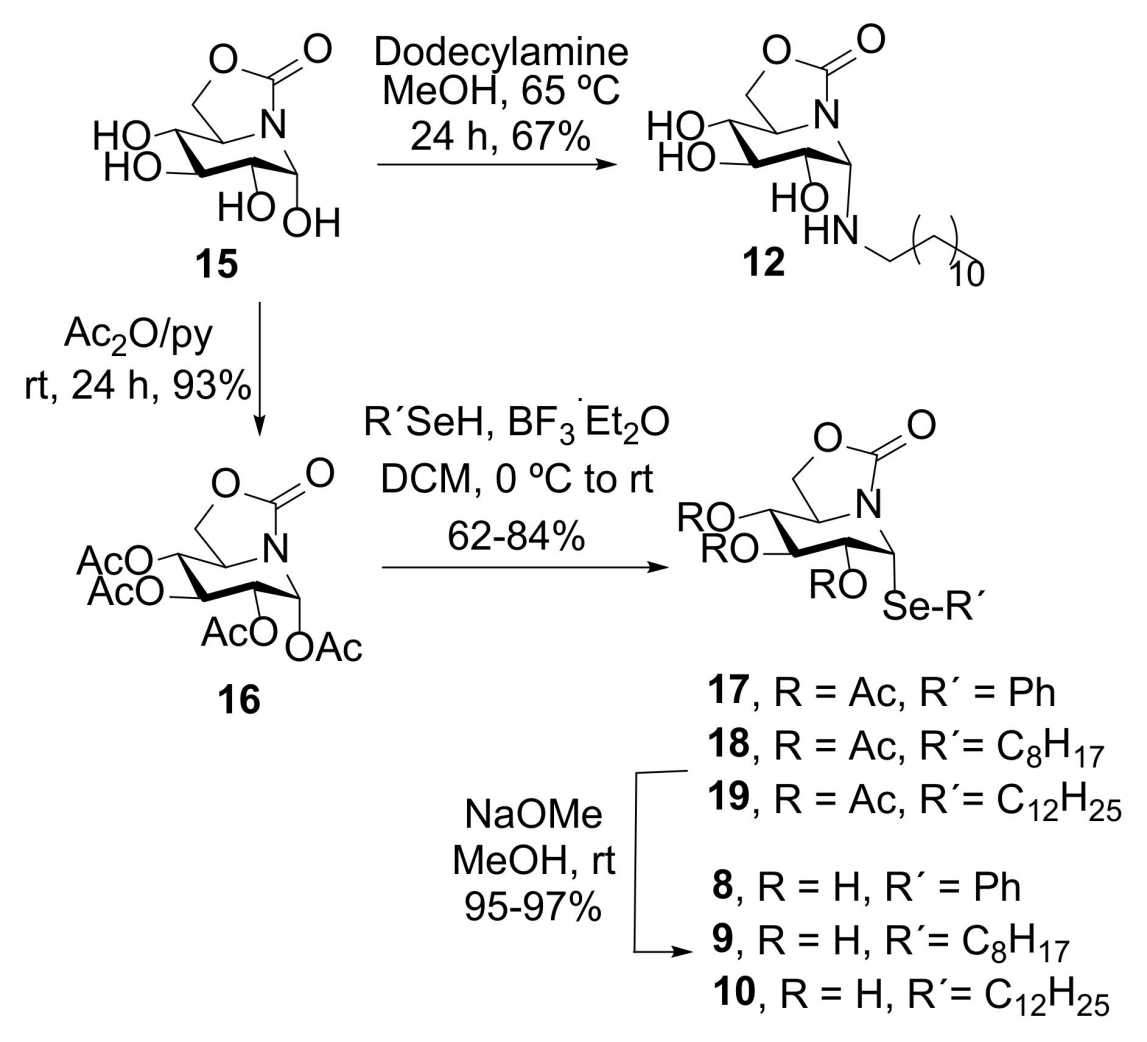
Synthesis of the novel pseudo-α-*N*-dodecyl (**12**) and *Se*-phenyl(octyl)(dodecyl) glycosides (**8**–**10**).

The α-phenyl(octyl)(dodecyl) ONJ pseudoanomeric selenides **8**–**10** were synthesized from (1*R*)-1,2,3,4-tetra-*O*-acetyl-5*N*,6*O*-oxomethylidenenojirimycin (**16**), accessible through an efficient synthetic scheme from d-glucuronolactone [56], by reaction with phenyl, octyl or docecyl selenol, respectively, in the presence of boron trifluoride etherate (BF_3_·Et_2_O) as glycosidation promotor (→ **17**–**19**) (Scheme 1). The aliphatic selenides were prepared from the corresponding commercially available bromoalkanes, elemental selenium and sodium borohydride [57]. Final conventional de-*O*-acetylation under Zemplén conditions afforded the target fully unprotected α-pseudoglycosyl selenides **8**–**10** (Scheme 1). This reaction scheme parallels that previously optimized for the synthesis of the *S*-linked analogues **13** [34] and **14** [51]. The *N*-dodecyl derivative **12** on its side was straightforward prepared following the reported experimental procedure used for the synthesis of the *N*-octyl sp^2^-IGL **11** [34]. *A solution* of reducing ONJ (**15**) and commercially available dodecylamine in methanol heated at 65 °C for 24 h led to the *gem*-diamine-type conjugate **12** in 67% yield after purification by column chromatography (Scheme 1). Only the α-anomer, with the lipid aglycone in axial orientation, was detected in the reaction mixture, even in this later case, which is amazing considering the overwhelming tendency of classical glycosylamines to enforce the equatorial disposition of the anomeric substituent by virtue of the reverse anomeric effect [58].

The structure of all compounds was confirmed by ^1^H, ^13^C and ^77^Se (for Se-derivatives), NMR, MS and combustion analysis. Note that all the *Se*-, *S*- and *N*-linked sp^2^-IGLs synthesized adopt a ^4^*C*_1_ chair type conformation with vicinal ^3^*J*_1,2_ coupling constants ranging between 4.8–6.0 Hz, characteristic of the α-anomeric configuration.

### 2.2. Antiproliferative and Antiparasitic Properties of the sp^2^-IGLs

**Figure 3:**
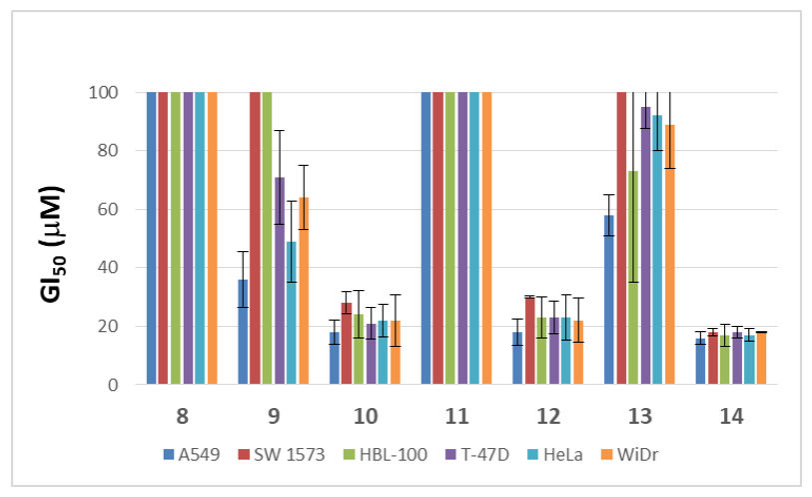
Comparison of the antiproliferative activity of sp^2^-IGLs **8**–**14** after evaluation by HTS. GI_50_ values and standard deviation (SD) from three independent experiments are depicted.

In order to assess whether the anticancer potential and the capabilities to inhibit parasite growth of this family of sp^2^-glycoconjugates are interrelated, in vitro evaluation of the antiproliferative and antiparasitic properties was conducted in parallel. High-throughput screening (HTS) against a panel of different human solid tumor cell lines including lung (A549, SW1573), breast (HBL-100, T-47D), cervix (HeLa) and colon (WiDr) cancer linages, helped us determine the antiproliferative potential of these sp^2^-IGLs. As for the antiparasitic activity, the growth inhibition of intracellular amastigotes of *Leishmania donovani* HU3 species, responsible for visceral Leishmaniasis (VL), was investigated. The results, expressed as the concentration to achieve 50% growth inhibition of tumor cells (GI_50_) and the concentration of compound that reduces cell growth by 50% versus untreated control cells (EC_50_) for arresting parasite development, are collected in Figure 3 and Table 1, respectively.

The critical effect of the lipid chain length on the antiproliferative activity was evident in the gem-diamine derivatives. The *N*-glycoside-type sp^2^-IGL **12**, bearing a dodecyl chain, showed GI_50_ values in the 18–30 μM range against all the cell lines tested, while the *N*-octyl derivative **11** was over the threshold in this assay (GI_50_ > 100 μM) in all cases. Although to a lesser extent, the same trend was encountered for *S*- and *Se*-sp^2^-IGLs. Thus, the dodecyl derivatives **14** and **10** displayed GI_50_ values of 16–28 μM, whereas the *S*- and *Se*-octyl counterparts, **13** and **9**, afforded GI_50_ values from 36 μM to >100 μM (Figure 3). The antiproliferative activity of the *N*-, *S*- and *Se*-dodecyl sp^2^-IGLs (**12**, **14** and **10**) is similar to that of the antineoplastic agent 5-fluorouracyl (5-FU) in the same assay (GI_50_ 5–50 μM) [9]. The utmost importance of the aliphatic lipid chain was further highlighted by the lack of activity of the aromatic *Se*-pseudoglycoside derivative **8**. Bearing in mind the variation of pH in tumor cells with respect to normal cells, specifically the acidity of the extracellular environment of a tumor tissue [59], stability experiments of selenoglycosides **8** and **9** at pH~4 were performed. The ^1^H-NMR spectra obtained after monitoring for 12 h in formate buffer evidenced their stability (See Supplementary Information).

Whilst in the dodecyl series the atom of the glycosidic linkage was essentially irrelevant for the antiproliferative potency, it was found to be a major determinant regarding the antiparasitic activity. Only the α-dodecylselenide **10** showed an EC_50_ value (13.42 ± 1.63 μM) for annihilation of the intracellular form of the protozoan parasite (amastigote forms) below the threshold (20 μM) in this assay (Table 1). Although this still implies an over one-order-of-magnitude lower efficacy as compared with the reference drug miltefosine (EC_50_ 0.44 ± 0.08 μM), it represents an interesting hit susceptible of optimization, given the versatility of the synthetic approach. Moreover, the toxicity profile against the monocytic and lung cell lines THP-1 and MRC-5, respectively, was not significantly different from that of miltefosine. Altogether, the data underline the α-dodecyl selenoglycoside **10** as a promising leishmanicidal agent in terms of potency and safety.

### 2.3. Anti-inflammatory Properties of the sp^2^-IGLs

The most active sp^2^-IGL in the tumor and parasite growth inhibition trials, namely the *Se*-dodecyl sp^2^-IGL **10**, was further assessed as an anti-inflammatory agent. Towards this end, its effect on nitrite production and on the expression of induced nitric oxide synthase (iNOS), major mediators of inflammation [60,61], under an inflammatory context has been investigated in comparison with the *S*-glycoside analogue **14**.

**Figure 4:**
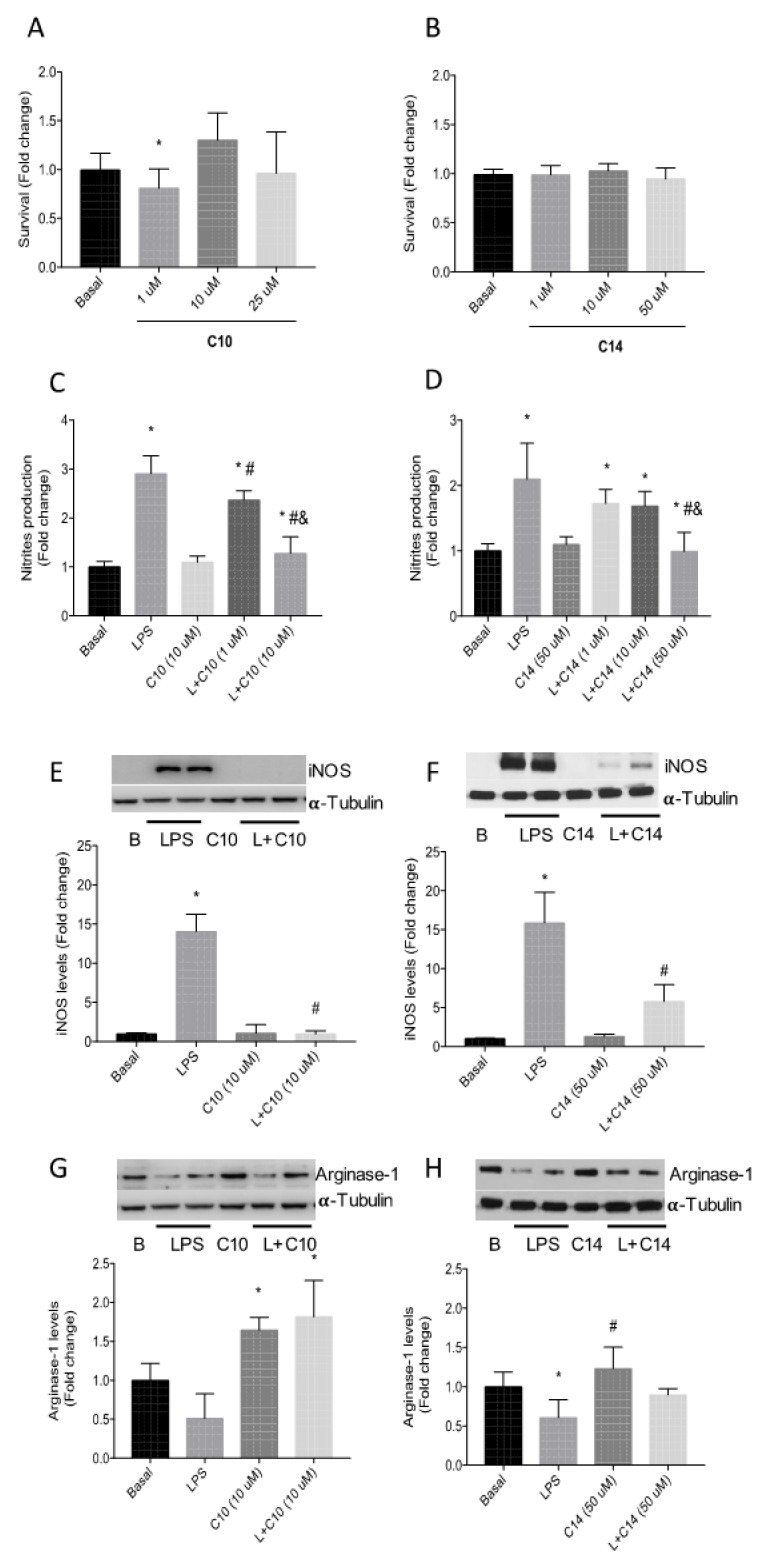
(**A**) Effect of *Se*-glycoside **10** and (**B**) *S*-glycoside **14** on the cellular viability (Bv.2 cells) determined by crystal violet staining. (**C**) Effect of **10** and (**D**) **14** on nitrite production. Bv.2 microglial cells were treated for 24 h with LPS (200 ng/mL) or LPS plus **10** (1–10 μM) and **14** (1–50 μM), respectively. Nitrite production was analyzed and related to the basal levels. Colorimetric quantification was performed. (**E**) Effect of **10** at 10 μM and (**F**) **14** at 50 μM on the expression of the LPS-induced iNOs protein. (**G**) Effect of **10** at 10 μM and (**H**) **14** at 50 μM on the levels of the anti-inflammatory marker arginase-1 in the absence or in the presence of LPS in Bv.2 microglial cells. In all cases, data represent mean ± S.E.M of fold changes relative to the basal values. * *p* ≤ 0.05 vs. Basal condition; # *p* ≤ 0.05 vs. LPS stimuli; and *p* ≤ 0.05 vs. L + C**10** (1 μM) or L + C**14** (10 μM) (two-way ANOVA followed by Bonferroni *t*-test).

Firstly, cell viability assays using a murine microglia Bv.2 cell line were performed after treatment with different concentrations of **14** and **10** for 24 h (Figure 4). As observed in Figure 4A,B, none of the concentrations used induced a negative effect on the cellular viability.

To gauge the effect of these compounds on nitrite production, Bv.2 cells were stimulated with bacterial lipopolysaccharide (LPS; 200 ng/mL), a pro-inflammatory stimulus, in the absence and in the presence of the *S*- and *Se*-sp^2^-IGLs (**14** and **10**), respectively, for 24 h. Both compounds exerted a dose-dependent reduction on nitrite production (Figure 4C,D), which was significantly more pronounced in the case of the selenide derivative **10** (basal level reached at 10 μM) as compared with the sulfide analogue **14** (basal level reached at 50 μM). Additionally, treatment with **10** at 10 μM fully abrogated the enhancement of iNOS elicited by LPS, whereas **14** at a five-fold higher concentration only reduced iNOS increase from 20-fold to about 8-fold (Figure 4E,F). Compound **10** at 10 μM also showed a direct effect on the induction of arginase-1, a marker of the classical anti-inflammatory response (M2) in microglia, either in the absence or in the presence of LPS (200 ng/mL; Figure 4G). However, treatment with **14** did not induce arginase-1 expression (Figure 4H). Taken together, these data accentuate the strong anti-inflammatory potential of selenium-linked sp^2^-IGLs.

## 3. Materials and Methods

### 3.1. General Methods

Reagents and solvents were purchased from commercial sources. ^1^H, ^13^C and ^77^Se NMR experiments were performed at 300, 75.5 and 95.4 MHz, respectively. 2-D COSY and HMQC experiments were carried out to assist on signal assignment. ^1^H-NMR-monitored kinetic evaluation of the stability of selenoglycosides (**8** and **9**) were performed at 500 MHz. For ESI mass spectra, 0.1 pM sample concentrations were used, mobile phase 50% aq MeCN at 0.1 mL min^−1^. Thin-layer chromatography was performed on precoated TLC plates, silica gel 30F-245, with visualization by UV light and by carrying with 0.2% *w*/*v* cerium (IV) sulphate-5% ammonium molybdate in 2 M H_2_SO_4_ or 0.1% ninhydrin in EtOH. Column chromatography was performed on Chromagel (silice 60 AC.C 70–200 μm). Optical rotations were measured with a JASCO P-2000 polarimeter, using a sodium lamp (λ = 589 nm) at 22 °C in 1 cm tube. All compounds were purified to ≥95% purity as determined by elemental microanalysis results obtained on a CHNSTruSpect Micro elemental analyzer (Instituto de Investigaciones Químicas de Sevilla, Spain) from vacuum-dried samples. The analytical results for C, H, N, and S were within ± 0.4 of the theoretical values. Deacetylation reactions were carried out by using a Zemplén procedure [62]. Addition of NaOMe (0.1 equiv/Ac mol) in MeOH at room temperature, followed by neutralization with solid CO_2_, evaporation of the solvent and purification by column chromatography. (1*R*)-5*N*,6*O*-Oxomethylidenenojirimycin (**15**) [56], (1*R*)-1,2,3,4-tetra-*O*-acetyl-5*N*,6*O*-oxomethylidenenojirimycin (**16**) [56], (1*S*)-1-amino-*N*-octyl-5*N*,6*O*-oxomethylidene-1-deoxynojirimycin (**11**) [34], (1*R*)-1-octylthio-5*N*,6*O*-oxomethylidenenojirimycin (**13**) [34], (1*R*)-1-dodecylthio-5*N*,6*O*-oxomethylidenenojirimycin (**14**) [51] were prepared according to previously reported procedures. Octyl selenol and dodecyl selenol were prepared from octyl bromide and dodecyl bromide, respectively, according to the literature procedure [57].

### 3.2. Synthesis of the sp^2^-IGLs

#### 3.2.1. Procedure for the Synthesis of Pseudo-*N*-Glycoside (**12**)

A solution of (1*R*)-5*N*,6*O*-oxomethylidenenojirimycin (**15**) (0.24 mmol) and the *n*-dodecylamine (0.24 mmol) in MeOH (3 mL) was heated at 65 °C for 24 h under Ar atmosphere. The solvent was eliminated under reduced pressure and the resulting residue was purified by column chromatography to afford the corresponding α-glycosylamine (**12**).

(1*S*)-1-Amino-*N*-dodecyl-5*N*,6*O*-oxomethylidene-1-deoxynojirimycin (**12**). Column chromatography (20:1 DCM-MeOH). Yield: 60 mg (67%). *R_f_* 0.69 (50:10:1 DCM-MeOH-H_2_O). [α]_D_ + 59.8 (*c* 0.9 in MeOH). ^1^H NMR (300 MHz, CD_3_OD): δ 4.63 (d, 1 H, *J*_1,2_ = 5.1 Hz, H-1), 4.48 (t, 1 H, *J*_6a,6b_ = *J*_5,6a_ = 8.5 Hz, H-6a), 4.27 (dd, 1 H, *J*_5,6b_ = 4.8 Hz, H-6b), 3.82 (ddd, 1 H, *J*_4,5_ = 9.5 Hz, H-5), 3.61 (t, 1 H, *J*_2,3_ = *J*_3,4_ = 9.5 Hz, H-3), 3.47 (dd, 1 H, H-2), 3.26 (t, 1 H, H-4), 2.63–2.53 (m, 2 H, NHC*H_2_*), 1.58–1.20 (m, 20 H, CH_2_), 0.90 (t, 3 H, ^3^*J*_H,H_ = 6.9 Hz, CH_3_). ^13^C NMR (75.5 MHz, CD_3_OD): δ 159.2 (CO), 75.6 (C-4), 74.5 (C-3), 72.3 (C-2), 69.6 (C-1), 67.9 (C-6), 54.6 (C-5), 47.5 (CH_2_NH), 33.1–23.7 (CH_2_), 14.4 (CH_3_). ESIMS: *m/z* 395.4 [M + Na]^+^. Anal. Calcd for C_19_H_36_N_2_O_5_: C 61.26, H 9.74, N 7.52. Found: C 61.14, H 9.58, N 7.33.

#### 3.2.2. General Procedure for the Synthesis of Pseudo-*Se*-Glycosides (**17**–**19**)

To a stirred solution of (1*R*)-1,2,3,4-tetra-*O*-acetyl-5*N*,6*O*-oxomethylidenenojirimycin (**16**) (150 mg, 0.40 mmol) in anhydrous DCM (8 mL) under Ar atmosphere, the corresponding selenol (0.84 mmol, 2.1 equiv.) and BF_3_.Et_2_O (0.18 mL, 1.41 mmol, 3.5 equiv.) were added at 0 °C. The mixture was stirred for 15 min, diluted with DCM (30 mL), washed with water (10 mL), saturated aqueous solution of NaHCO_3_ (2 × 10 mL) and water (10 mL), dried (MgSO_4_), filtered and concentrated to afford the corresponding per-*O*-acetylated selenoglycosides. The pure α-anomer O-acetylated intermediates (**17**–**19**) were obtained after purification by column chromatography using the solvents indicated in each case.

(1*R*)-2,3,4-Tri-*O*-acetyl-1-phenylseleno-5*N*,6*O*-oxomethylidenenojirimycin (**17**). Column chromatography (1:2 EtOAc-cyclohexane). Yield: 53 mg (84%). *R_f_* 0.78 (2:1 EtOAc-cyclohexane). [α]_D_ + 142.7 (*c* 1.1 in DCM). ^1^H NMR (300 MHz, CDCl_3_) δ 7.70–7.25 (m, 5 H, Ph), 6.10 (d, 1 H, *J*_1,2_ = 5.7 Hz, H-1), 5.60 (t, 1 H, *J*_2,3_ = *J*_3,4_ = 9.6 Hz, H-3), 5.03 (dd, 1 H, H-2), 4.95 (t, 1 H, *J*_4,5_ = 9.6 Hz, H-4), 4.29–4.04 (m, 3 H, H-5, H-6a, H-6b), 2.09–2.06 (3 s, 9 H, MeCO). ^13^C NMR (75.5 MHz, CDCl_3_) δ 169.9–169.3 (Me*CO*), 154.2 (CO), 136.1–125.2 (Ph), 72.3 (C-4), 70.7 (C-2, C-3), 65.6 (C-6), 54.7 (C-1), 51.9 (C-5), 20.5 (*Me*CO). ^77^Se NMR (95.4 MHz, CDCl_3_) δ 347.1. ESIMS: *m/z* 494.1 [M + Na]^+^. Anal. Calcd for C_19_H_21_NO_8_Se: C 48.52, H 4.50, N 2.98. Found: C 48.61, H 4.57, N 2.84.

(1*R*)-2,3,4-Tri-*O*-acetyl-1-octylseleno-5*N*,6*O*-oxomethylidenenojirimycin (**18**). Column chromatography (1:3 EtOAc-cyclohexane). Yield: 150 mg (74%). *R_f_* 0.85 (2:1 EtOAc-cyclohexane). [α]_D_ + 93.3 (*c* 1.1 in DCM). ^1^H NMR (300 MHz, CDCl_3_) δ 5.88 (d, 1 H, *J*_1,2_ = 6.0 Hz, H-1), 5.35 (t, 1 H, *J*_2,3_ = *J*_3,4_ = 9.6 Hz, H-3), 4.93 (t, 1 H, *J*_4,5_ = 9.6 Hz, H-4), 4.86 (dd, 1 H, H-2), 4.42 (t, 1 H, *J*_6a,6b_ = *J*_5,6a_ = 9.0 Hz, H-6a), 4.25 (dd, 1 H, *J*_5,6b_ = 7.0 Hz, H-6b), 4.11 (td, 1 H, H-5), 2.66 (ddd, 1 H, ^2^*J*_H,H_ = 12.0 Hz, ^3^*J*_H,H_ = 8.1 Hz, ^3^*J*_H,H_ = 6.6 Hz, SeCH_2_), 2.51 (ddd, 1 H, SeCH_2_), 2.05–2.00 (3 s, 9 H, MeCO), 1.70–1.50 (m, 2 H, SeCH_2_C*H_2_*), 1.37–1.17 (m, 10 H, CH_2_), 0.84 (t, 3 H, ^3^*J*_H,H_ = 7.0 Hz, CH_3_). ^13^C NMR (75.5 MHz, CDCl_3_) δ 170.0–169.4 (Me*CO*), 155.0 (CO), 72.5 (C-4), 70.5 (C-2, C-3), 66.1 (C-6), 51.8 (C-5), 51.2 (C-1), 31.7–22.6 (CH_2_), 20.7–20.5 (*Me*CO), 14.1 (CH_3_). ^77^Se NMR (95.4 MHz, CDCl_3_) δ 219.9. ESIMS: *m/z* 530.2 [M + Na]^+^. Anal. Calcd for C_21_H_33_NO_8_Se: C 49.80, H 6.57, N 2.77. Found: C 49.96, H 6.69, N 2.72.

(1*R*)-2,3,4-Tri-*O*-acetyl-1-dodecylseleno-5*N*,6*O*-oxomethylidenenojirimycin (**19**). Column chromatography (1:2 EtOAc-cyclohexane). Yield: 140 mg (62%). *R_f_* 0.57 (1:1 EtOAc-cyclohexane). [α]_D_ + 82.8 (*c* 1.0 in DCM). ^1^H NMR (300 MHz, CDCl_3_) δ 5.91 (d, 1 H, *J*_1,2_ = 6.0 Hz, H-1), 5.38 (t, 1 H, *J*_2,3_ = *J*_3,4_ = 9.6 Hz, H-3), 4.92 (t, 1 H, *J*_4,5_ = 9.6 Hz, H-4), 4.86 (dd, 1 H, H-2), 4.44 (t, 1 H, *J*_6a,6b_ = *J*_5,6a_ = 9.0 Hz, H-6a), 4.27 (dd, 1 H, *J*_5,6b_ = 9.0 Hz, H-6b), 4.14 (ddd, 1 H, H-5), 2.68 (ddd, 1 H, ^2^*J*_H,H_ = 12.0 Hz, ^3^*J*_H,H_ = 8.4 Hz, ^3^*J*_H,H_ = 6.6 Hz, SeCH_2_), 2.54 (ddd, 1 H, SeCH_2_), 2.07–2.02 (3 s, 9 H, MeCO), 1.70–1.20 (m, 20 H, CH_2_), 0.87 (t, 3 H, ^3^*J*_H,H_ = 7.0 Hz, CH_3_). ^13^C NMR (75.5 MHz, CDCl_3_) δ 170.0–169.4 (Me*CO*), 155.0 (CO), 72.5 (C-4), 70.6–70.5 (C-2, C-3), 66.1 (C-6), 51.9 (C-5), 51.3 (C-1), 31.9–29.0 (CH_2_), 24.0 (SeCH_2_), 20.7–20.5 (*Me*CO), 14.1 (CH_3_). ^77^Se NMR (95.4 MHz, CDCl_3_) δ 219.9. ESIMS: *m/z* 586.3 [M + Na]^+^. Anal. Calcd for C_25_H_41_NO_8_Se: C 53.38, H 7.35, N 2.49. Found: C 53.42, H 7.41, N 2.39.

#### 3.2.3. General Procedure for the Synthesis of the Deprotected Pseudo-*Se*-Glycosides (**8**–**10**)

To a stirred solution of the corresponding acetylated pseudo-*Se*-glycoside (0.20 mmol) in MeOH (4 mL), NaOMe 1 M (0.06 mmol) was added and the reaction mixture was stirred at room temperature for 30–60 min. Neutralization with solid CO_2_, evaporation of the solvent and purification by column chromatography using the solvents indicated in each case yielded the fully deprotected selenoglycosides **8**–**10**.

(1*R*)-1-Phenylseleno-5*N*,6*O*-oxomethylidenenojirimycin (**8**). Column chromatography (9:1 EtOAc-MeOH). Yield: 21 mg (95%). *R_f_* 0.61 (9:1 EtOAc-MeOH). [α]_D_ + 228.5 (*c* 0.8 in MeOH). ^1^H NMR (300 MHz, CD_3_OD) δ 7.70–7.20 (m, 5 H, Ph), 5.80 (d, 1 H, *J*_1,2_ = 4.8 Hz, H-1), 4.29 (t, 1 H, *J*_6a,6b_ = *J*_5,6a_ = 9.0 Hz, H-6a), 4.17 (dd, 1 H, *J*_5,6b_ = 5.7 Hz, H-6b), 3.89 (td, 1 H, *J*_4,5_ = 9.4 Hz, H-5), 3.75–3.63 (m, 2 H, H-2, H-3), 3.38–3.31 (m, 1 H, H-4). ^13^C NMR (75.5 MHz, CD_3_OD) δ 157.4 (CO), 134.6–127.7 (Ph), 76.1–72.9 (C-2, C-3, C-4), 67.6 (C-6), 60.2 (C-1), 54.8 (C-5). ^77^Se NMR (95.4 MHz, CD_3_OD) δ 336.2. ESIMS: *m/z* 368.1 [M + Na]^+^. Anal. Calcd for C_13_H_15_NO_5_Se: C 45.36, H 4.39, N 4.07. Found: C 45.12, H 4.27, N 3.89.

(1*R*)-1-Octylseleno-5*N*,6*O*-oxomethylidenenojirimycin (**9**). Column chromatography (6:1 EtOAc-MeOH). Yield: 72.4 mg (95%). *R_f_* 0.73 (1:4 MeOH-EtOAc). [α]_D_ + 130.1 (*c* 1.1 in MeOH). ^1^H NMR (300 MHz, CD_3_OD) δ 5.56 (d, 1 H, *J*_1,2_ = 4.8 Hz, H-1), 4.57 (t, 1 H, *J*_6a,6b_ = *J*_5,6a_ = 9.0 Hz, H-6a), 4.28 (dd, 1 H, *J*_5,6b_ = 6.3 Hz, H-6b), 3.92 (td, 1 H, *J*_4,5_ = 9.0 Hz, H-5), 3.63–3.48 (m, 2 H, H-2, H-3), 3.36 (t, 1 H, *J*_3,4_ = 9.0 Hz, H-4), 2.66–2.47 (m, 2 H, SeCH_2_), 1.78–1.57 (m, 2 H, SeCH_2_C*H_2_*), 1.45–1.24 (m, 10 H, CH_2_), 0.90 (t, 3 H, ^3^*J*_H,H_ = 7.0 Hz, CH_3_). ^13^C NMR (75.5 MHz, CD_3_OD) δ 157.9 (CO), 76.0–75.4 (C-3, C-4), 72.6 (C-2), 68.1 (C-6), 56.4 (C-1), 54.9 (C-5), 32.9–23.5 (CH_2_), 14.4 (CH_3_). ^77^Se NMR (95.4 MHz, CD_3_OD) δ 204.4. ESIMS: *m/z* 404.2 [M + Na]^+^. Anal. Calcd for C_15_H_27_NO_5_Se: C 47.37, H 7.16, N 3.68, Se 20.76. Found: C 47.17, H 7.15, N 3.53.

(1*R*)-1-Dodecylseleno-5*N*,6*O*-oxomethylidenenojirimycin (**10**). Column chromatography (6:1 EtOAc-MeOH). Yield: 30 mg (97%). *R_f_* 0.61 (6:1 EtOAc- MeOH). [α]_D_ + 124.5 (*c* 0.9 in MeOH). ^1^H NMR (300 MHz, CD_3_OD) δ 5.55 (d, 1 H, *J*_1,2_ = 5.1 Hz, H-1), 4.57 (t, 1 H, *J*_6a,6b_ = *J*_5,6a_ = 9.0 Hz, H-6a), 4.28 (dd, 1 H, *J*_5,6b_ = 6.0 Hz, H-6b), 3.92 (td, 1 H, *J*_4,5_ = 9.0 Hz, H-5), 3.60–3.48 (m, 2 H, H-2, H-3), 3.40–3.31 (m, 1 H, H-4), 2.66–2.46 (m, 2 H, SeCH_2_), 1.80–1.60 (m, 2 H, SeCH_2_C*H_2_*), 1.45–1.20 (m, 18 H, CH_2_), 0.90 (t, 3 H, ^3^*J*_H,H_ = 6.9 Hz, CH_3_). ^13^C NMR (75.5 MHz, CD_3_OD) δ 158.0 (CO), 76.0–75.5 (C-3, C-4), 72.7 (C-2), 68.2 (C-6), 56.5 (C-1), 54.9 (C-5), 33.0–23.5 (CH_2_), 14.4 (CH_3_). ^77^Se NMR (95.4 MHz, CD_3_OD) δ 204.4. ESIMS: *m/z* 460.2 [M + Na]^+^. Anal. Calcd for C_19_H_35_NO_5_Se: C 52.29, H 8.08, N 3.21. Found: C 52.28, H 8.35, N 3.18.

### 3.3. Procedure for Antiproliferative Assays

All reagents were used as purchased from commercial suppliers without further purification. The human solid tumor cell lines used in this study were: A549, SW1573 (lung), HBL-100, T-47D (breast), HeLa (cervix) and WiDr (colon). These cell lines were a kind gift from Prof. G.J. Peters (VU Medical Center, Amsterdam, The Netherlands).

#### Chemosensitive Testing

Cells were inoculated onto 96-well microtiter plates in a volume of 100 μL per well at densities of 2500 (A549, HBL-100, HeLa, and SW1573) and 5000 (T-47D and WiDr) cells per well, based on their doubling times. Compounds were initially dissolved in DMSO at 400 times the desired final maximum test concentration. Control cells were exposed to an equivalent concentration of DMSO (0.25% *v*/*v*, negative control). Each agent was tested in triplicate at different dilutions in the range of 1–100 μM. The drug treatment started on day 1 after plating. Drug incubation times were 48 h, after which cells were precipitated with 25 μL ice-cold TCA (50% *w*/*v*) and fixed for 60 min at 4 °C. Then the SRB assay was performed. The optical density (OD) of each well was measured at 530 nm, using BioTek’s PowerWave XS Absorbance Microplate Reader. Values were corrected for background OD from wells only containing medium. The antiproliferative activity for each compound, expressed as GI_50_ values, was calculated according to NCI formulas [63].

### 3.4. Procedure for Antileishmanial Assays

#### 3.4.1. Reagents

For the biological assays, stock solutions of the synthesized compounds in DMSO at 10 mM were prepared. 3-(4,5-Dimethyltriazol-2-yl)-2,5-diphenyltetrazolium bromide (MTT) and phorbol 12-myristate 13-acetate (PMA) were purchased from Sigma-Aldrich (St. Louis, MO, USA). Miltefosine was purchased from Zentaris GmbH (Frankfurt am Main, Germany). Hygromycin B was purchased from Invitrogen (Carlsbad, CA, USA). Kit Luciferase Assay System was purchased from Promega. L-glutamine and penicillin/streptomycin were obtained from Gibco.

#### 3.4.2. Cell Lines Culture and Determination of Cellular Toxicity

Human myelomonocytic cell line THP-1 was grown at 37 °C and 5% CO_2_ in RPMI-1640 supplemented with 10% iFBS, 2 mM glutamate, 100 U/mL penicillin and 100 μg/mL streptomycin. 3 × 10^4^ cells/well in 96-well plates were differentiated to macrophages with 20 ng/mL of PMA treatment for 48 h followed by 24 h of culture in fresh medium [64]. MRC5 cells were grown at 37 °C and 5% CO_2_ in DMEM complete media. 4000 cells/well in 96-well plates were incubated for 24 h at 37 °C and 5% CO_2_.

Cellular toxicity of all compounds was determined using the colorimetric MTT-based assay after incubation at 37 °C for 72 h in the presence of increasing concentrations of compounds (final maximal concentrations 200 µM) [65]. The results are expressed as EC_50_ values, as the concentration of the compound that reduce cell growth by 50% versus untreated control cells. Assays were performed in triplicate.

#### 3.4.3. Susceptibility Analysis in Intracellular *Leishmania* Amastigotes

Macrophage-differentiated-THP-1 cells, which are considered a suitable model for human macrophages, were plated at a density of 3 × 10^4^ macrophages/well in 96-well white polystyrene microplates, and were infected at a macrophage/parasite ratio of 1:10 with *L. donovani* HU3 promastigotes. 24 h after infection at 35 °C and 5% CO_2_, extracellular parasites were removed by washing with serum-free medium. Infected cell cultures were then incubated at different compound concentrations in RPMI 1640 medium plus 10% iFBS at 37 °C with 5% CO_2_ for 72 h. To determine the susceptibility of *L. donovani*-LUC amastigotes, infected macrophages maintained in 96-well plates were lysed and then luminescence intensity was measured as indicative of the intracellular parasite growth, using the Luciferase Assay System Kit (Promega, Madison, WI, USA) according to the instructions of the supplier.

### 3.5. Procedure for Anti-inflammatory Assays

#### 3.5.1. Reagents

Fetal bovine serum (FBS) and culture media were obtained from Invitrogen (Grand Island, NY, USA). Bovine serum albumin (BSA), crystal violet and bacterial lipopolysaccharide (LPS) were purchased from Sigma-Aldrich (St Louis, MO, USA). Bradford reagent, acrylamide, immunoblot PVDF membranes and chemiluminiscent HRP Substrate were purchased from Bio-Rad (Madrid, Spain).

#### 3.5.2. Antibodies

Antibodies against iNOS (ab 15323) were purchased from Abcam (Cambridge, UK). Anti-arginase-1 (BD610708) antibody was purchased from BD Bioscience (Madrid, Spain), and anti-α-tubulin (T-5168) antibody was from Sigma-Aldrich (St Louis, MO, USA).

#### 3.5.3. Cell Culture

Mouse microglia Bv.2 cell line was supplied by Dr. M.L. Nieto (IBGM, Valladolid, Spain). Bv.2 cells were cultured at 37 °C in a humidified atmosphere with 5% CO_2_ in RPMI supplemented with 10% (*v*/*v*) heat-inactivated FBS, 1% (*v*/*v*) penicillin/streptomycin (Sigma) and 2 mM L-glutamine (Gibco, Carlsbad, CA, USA). Cells were grown up to 70% confluence and then washed twice with PBS and further cultured in serum-free medium and stimulated with LPS (200 ng/mL) with or without **14**/**10** (0.1–50 µM) for 24 h.

#### 3.5.4. Analysis of the Cellular Viability by Crystal Violet Staining

After cell treatments, the medium was discarded, and the remaining viable adherent cells were stained with crystal violet (0.2% *w*/*v* in 2% ethanol) for 20 min. After this time, plates were rinsed with tap water and allowed to dry, and 1% SDS was added to solubilize them. The absorbance of each plate was read spectrophotometrically at 560 nm.

#### 3.5.5. Analysis of Nitrites (NO_2_^−^)

Nitrite levels were measured by using the Griess method [66]. Briefly, nitrites turn into a pink compound in contact with an acid solution containing 1% sulphanilamide and 0.1% N-(1-naphthyl)ethylenediamine (NEDA), and can be quantified by a colorimetric method at 540 nm in a microplate reader (Versamax Tunable Microplate reader, Molecular Devices, Sunnyvaley, CA, USA).

#### 3.5.6. Western Blot

Bv.2 microglial cells were homogenized in lysis buffer containing 50 mM Tris-HCl, pH 7.4, 150 mM NaCl, 1 mM Na_3_VO_4_, 1 mM NaF, 1 mM EGTA, 15% (*w*/*v*) NP40 and 0.25% (*w*/*v*) sodium deoxycholate, supplemented with protease inhibitors (10 µg/mL leupeptin, 10 µg/mL aprotinin, and 100 µg/mL phenylmethylsulphonyl fluoride). All debris was removed by centrifugation at 14,000× *g* for 10 min at 4 °C and protein concentration was quantified using the Bio-Rad protein assay with BSA as a standard. Equivalent amounts of protein were resolved using denaturing sodium dodecyl sulphate-polyacrylamide gel electrophoresis (SDS-PAGE), followed by transfer to PVDF membranes (Bio-Rad). Membranes were blocked using 5% non-fat dried milk or 3% BSA in 10 mM Tris-HCl, 150 mM NaCl, pH 7.5 (TBS), and were incubated overnight with several antibodies (1:2000 unless otherwise stated) in a chemiluminescence reagent (Bio-Rad).

#### 3.5.7. Statistical Analysis

Densitometry of the Western blots was performed using the ImageJ program. Values in all graphs represented the mean ± SEM. Statistical tests were performed using SPSS 21.0 for Windows (SPSS Inc. IBM, Armonk, NY, USA). Data were analyzed by one-way ANOVA followed by Bonferroni t-test or by paired t-test when comparisons were among two groups. Differences were considered significant at *#& *p* ≤ 0.05.

## 4. Conclusions

In summary, an efficient synthesis of glycolipid mimetics featuring a bicyclic sp^2^-iminosugar aglycone and a α-*Se*-linked lipid aglycone, namely *Se*-sp^2^-IGLs, has been implemented. Similarly to the previously reported *N*- and *S*-sp^2^-IGLs, the antiproliferative activity of the novel selenoglycosides is strongly dependent on the lipid tail length, the *Se*-dodecyl derivative **10** being clearly superior to the *Se*-octyl derivative **9**, whereas the *Se*-phenyl analogue **8** was, as anticipated, inactive. Most interestingly, compound **10** proved superior to the thioglycoside counterpart **14** at arresting *Leishmania* amastigote growth and counteracting the inflammatory response in mouse microglia upon LPS challenge. The ensemble of results supports the hypothesis of these compounds behaving as multitarget drugs for immune-related conditions. Further studies aiming at identifying the molecular basis of the biological activity of **10** and the development of candidates featuring improved capabilities are currently pursued in our laboratories.

## Figures and Tables

**Table 1:** EC_50_ (μM) values of sp^2^-IGLs **8–14** against intracellular amastigote forms of *Leishmania donovani* HU3 and cytotoxicity profile in THP-1 and MRC-5 cells ^1^.

Compound	Intracellular Amastigotes HU3	THP-1 Cell Line	MRC-5 Cell Line
**8** (Se-Ph)	>20	>200	172.16 ± 27.02
**9** (Se-C_8_)	>20	>200	94.31 ± 4.95
**10** (Se-C_12_)	13.42 ± 1.63	84.25 ± 0.06	31.22 ± 1.61
**11** (NH-C_8_)	>20	208.69 ± 10.32	154.52 ± 5.30
**12** (NH-C_12_)	>20	122.50 ± 13.30	49.60 ± 4.20
**13** (S-C_8_)	>20	122.33 ± 5.79	58.14 ± 12.62
**14** (S-C_12_)	>20	70.46 ± 3.75	43.07 ± 9.83
Miltefosine	0.44 ± 0.08	26.86 ± 3.08	57.58 ± 6.38

^1^ Data are means of EC_50_ ± SD from three independent experiments.

## Data Availability

The data used to support the findings of this article are available from the corresponding author.

## Supplementary Materials

Supplementary data to this article (Figures S1–S13: NMR spectra for all new compounds **8**, **9**, **10**, **12**, **17**, **18** and **19**; Figures S14–S17: ^1^H-NMR-monitored kinetic evaluation of the stability of **8** and **9** at pH 4.4 and 4.6, respectively). Table S1: GI_50_ values (μM) of the sp^2^-IGLs (**8**–**14**) tested as antiproliferative agents by HTS.

## Sample Availability

Samples of the new compounds are available from the authors.
