# Supplementary material for: Synthesis of sp2-Iminosugar Selenoglycolipids as Multitarget Drug Candidates with Antiproliferative, Leishmanicidal and Anti-Inflammatory Properties
Source: Molecules. 2021 Dec 11;26(24):7501. doi: 10.3390/molecules26247501 (PMC8705409; doi:10.3390/molecules26247501)
Supplement: Supplementary file 1 [file molecules-26-07501-s001.zip › molecules-1473803-supplementary.pdf]

molecules

MDPI

*Supporting Information*

# Synthesis of sp²-iminosugar selenoglycolipids as multitarget drug candidates with antiproliferative, leishmanicidal and anti-inflammatory properties

**Elena M. Sánchez-Fernández [1,*], Raquel García-Hernández [2], Francisco Gamarro[2], Ana I. Arroba [3], Manuel Aguilar-Diosdado [3], José M. Padrón [4], José M. García Fernández [5] and Carmen Ortiz Mellet [1]**

[1] Department of Organic Chemistry, Faculty of Chemistry, University of Seville, C/ Profesor García González 1, 41012 Seville, Spain
[2] Instituto de Parasitología y Biomedicina "López-Neyra", IPBLN-CSIC, Parque Tecnológico de Ciencias de la Salud, 18016-Granada, Spain
[3] Biomedical Research and Innovation Institute of Cádiz (INiBICA) Research Unit, Puerta del Mar University Hospital, Av/ Ana de Viya 21, 11009 Cádiz, Spain
[4] BioLab, Instituto Universitario de Bio-Orgánica Antonio González (IUBO AG), Centro de Investigaciones Biomédicas de Canarias (CIBCAN), Universidad de La Laguna, 38206, La Laguna, Spain
[5] Instituto de Investigaciones Químicas (IIQ), CSIC - University of Sevilla, Américo Vespucio 49, 41092 Sevilla, Spain
* Correspondence: author: Elena M. Sánchez Fernández, esanchez4@us.es

**Table of Contents:**

NMR spectra of new sp²-IGLs (**8**, **9**, **10**, **12**, **17**, **18**, **19**)

$^1$H-NMR-monitored kinetic evaluation of the stability of compounds **8** and **9** at pH 4.4 and 4.6, respectively.

Table S1 ($GI_{50}$ values of **8-14**)

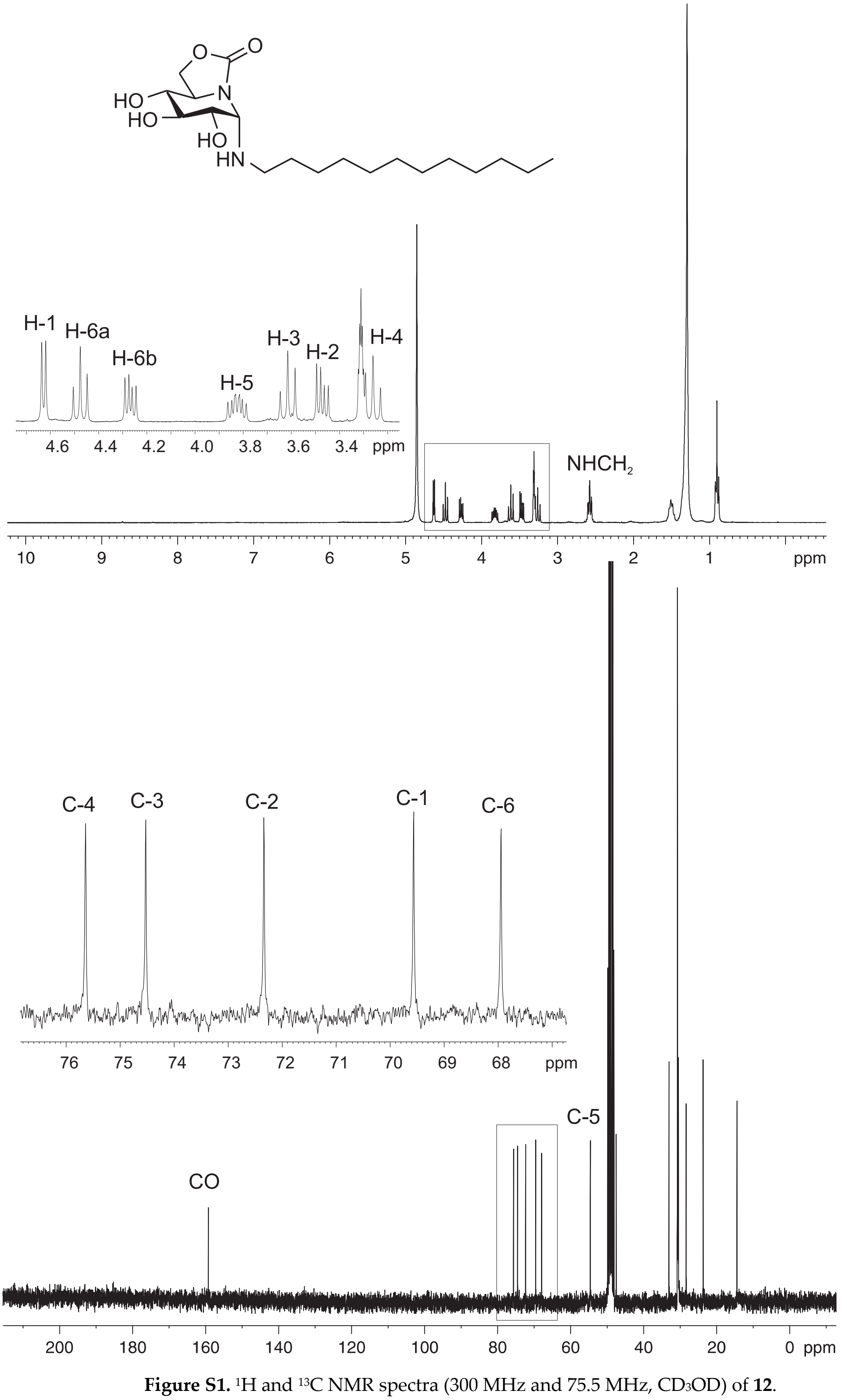


**Figure S1.** $^{1}H$ and $^{13}C$ NMR spectra (300 MHz and 75.5 MHz, $CD_3OD$) of **12**.

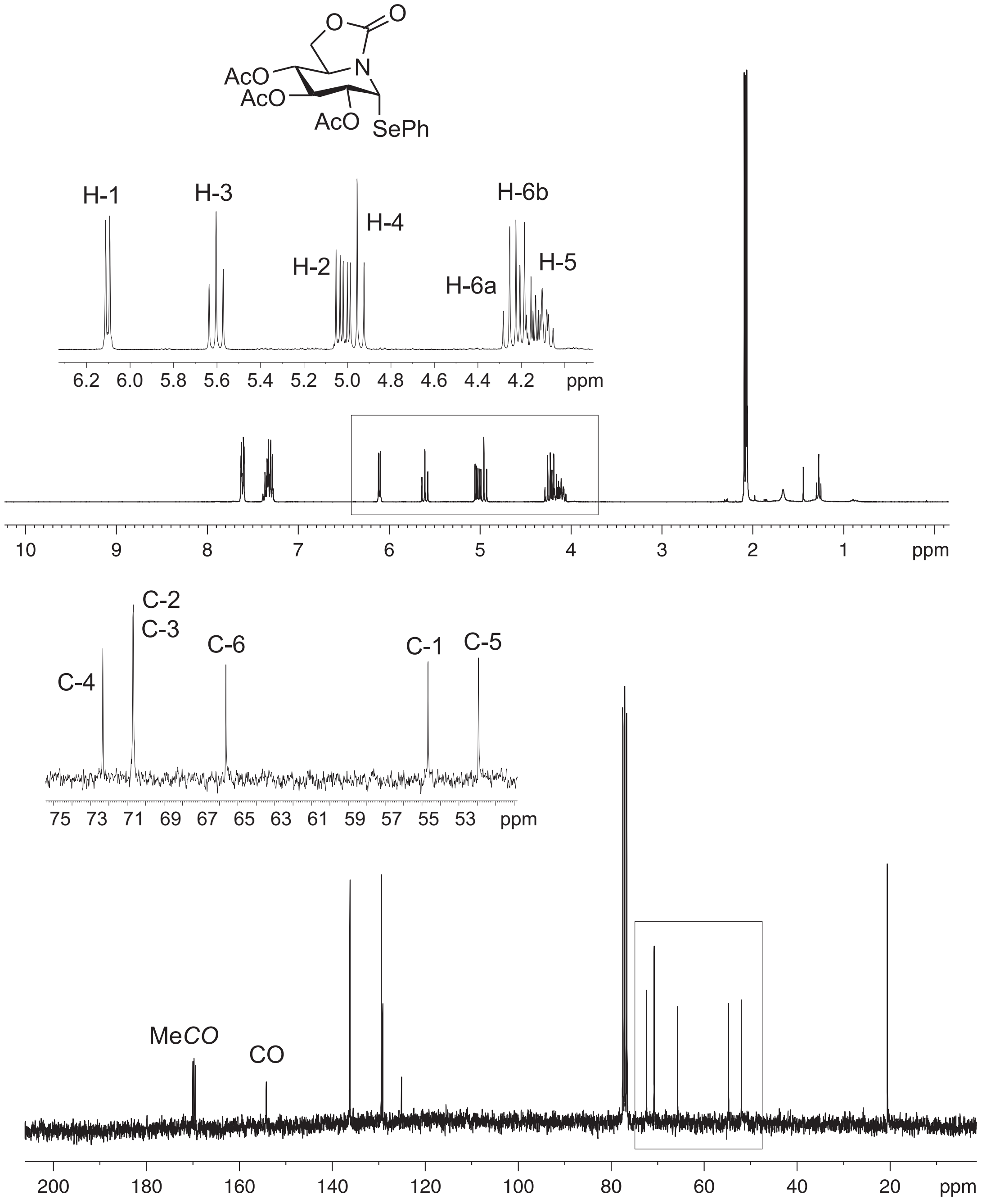


**Figure S2.** $^{1}H$ and $^{13}C$ NMR spectra (300 MHz and 75.5 MHz, $CDCl_3$) of **17**.

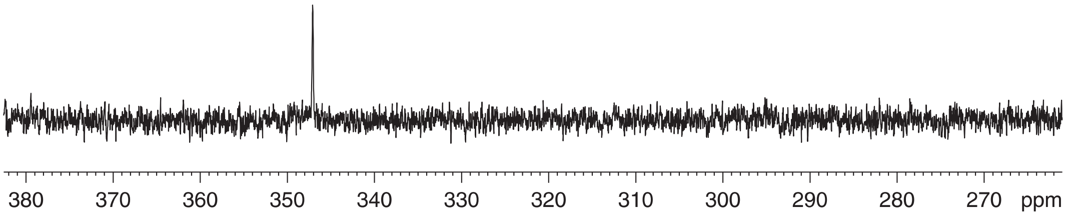


**Figure S3.** $^{77}Se$ NMR spectrum (95.4 MHz, $CDCl_3$) of **17**.

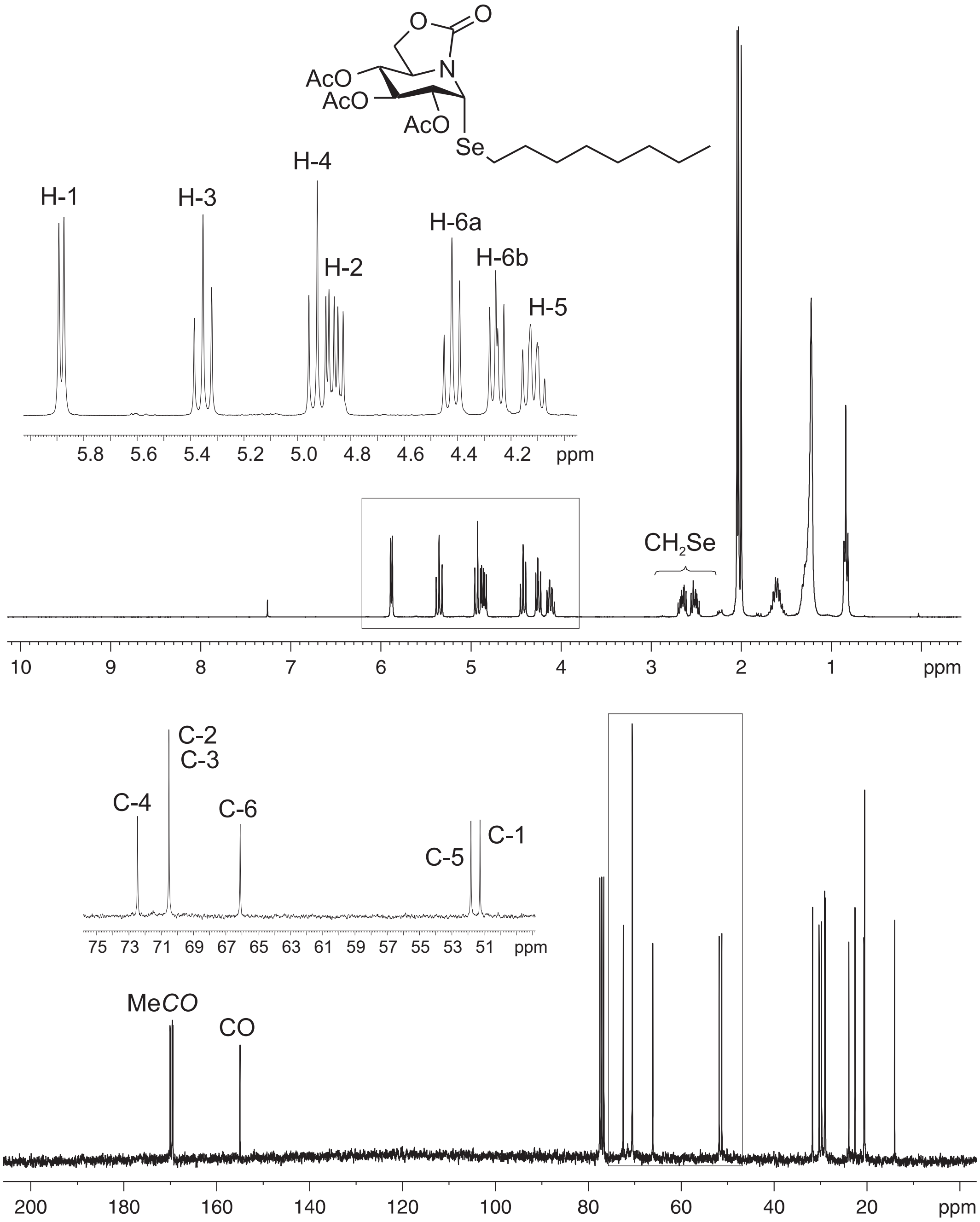


**Figure S4.** $^{1}H$ and $^{13}C$ NMR spectra (300 MHz and 75.5 MHz, $CDCl_3$) of **18**.

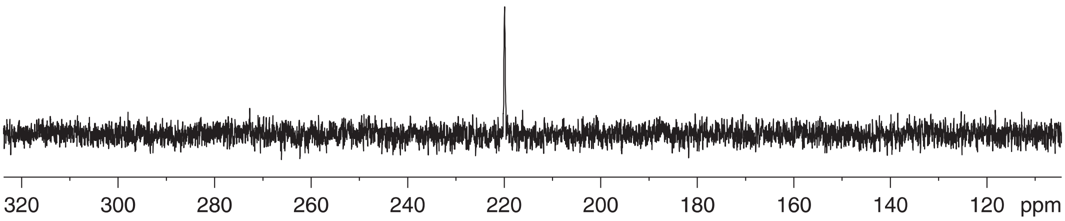


**Figure S5.** $^{77}Se$ NMR spectrum (95.4 MHz, $CDCl_3$) of **18**.

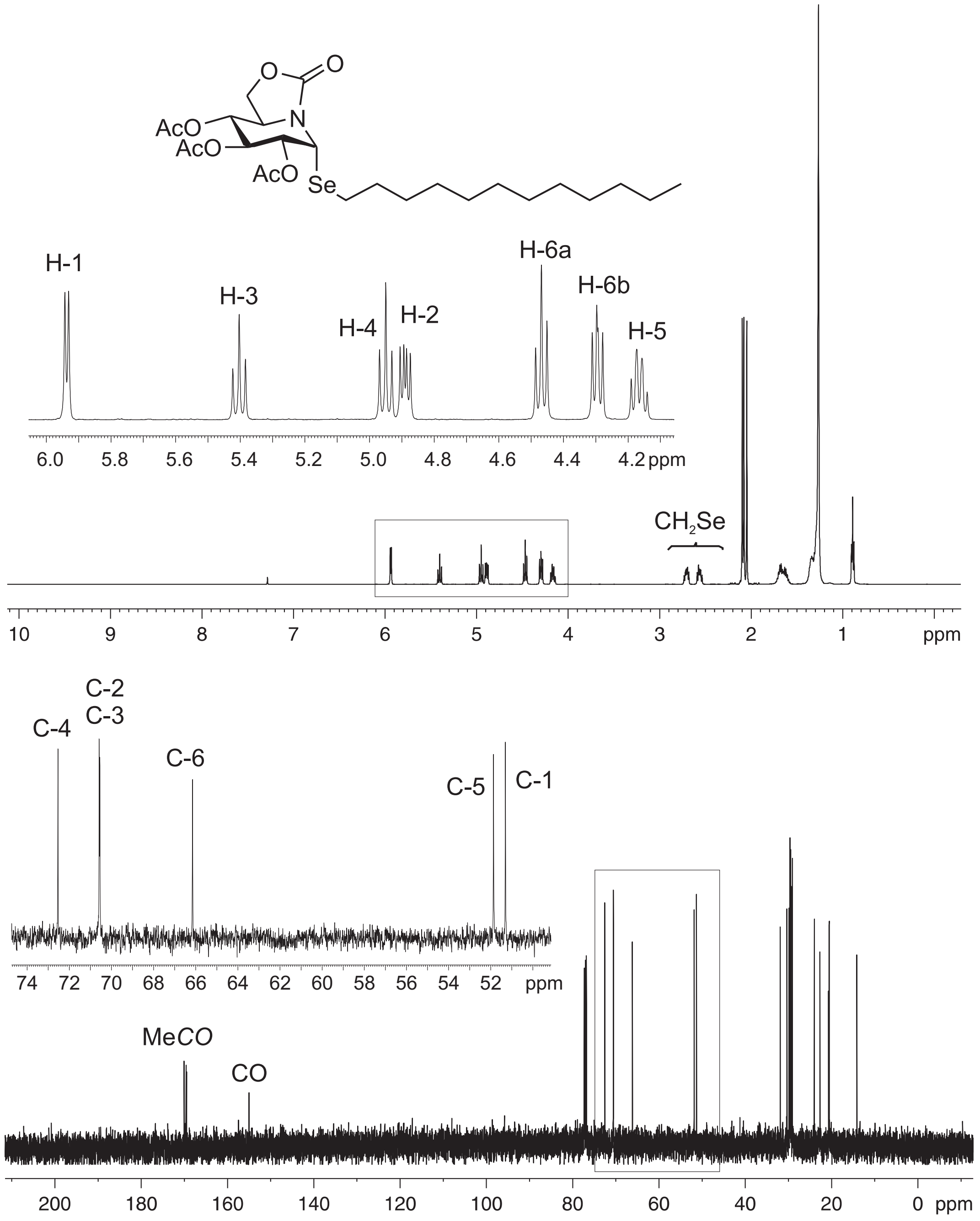


**Figure S6.** $^{1}H$ and $^{13}C$ NMR spectra (300 MHz and 75.5 MHz, $CDCl_3$) of **19**.

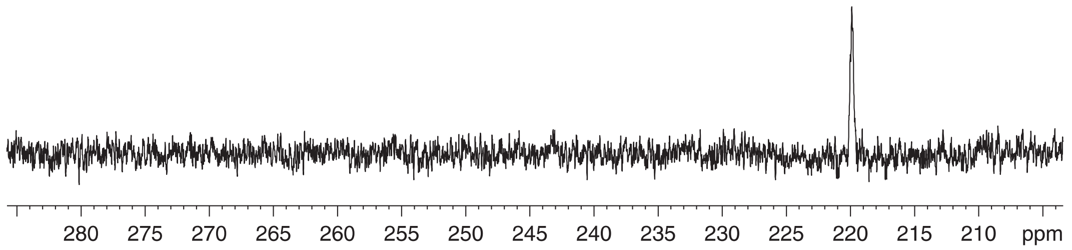


**Figure S7.** $^{77}Se$ NMR spectrum (95.4 MHz, $CDCl_3$) of **19**.

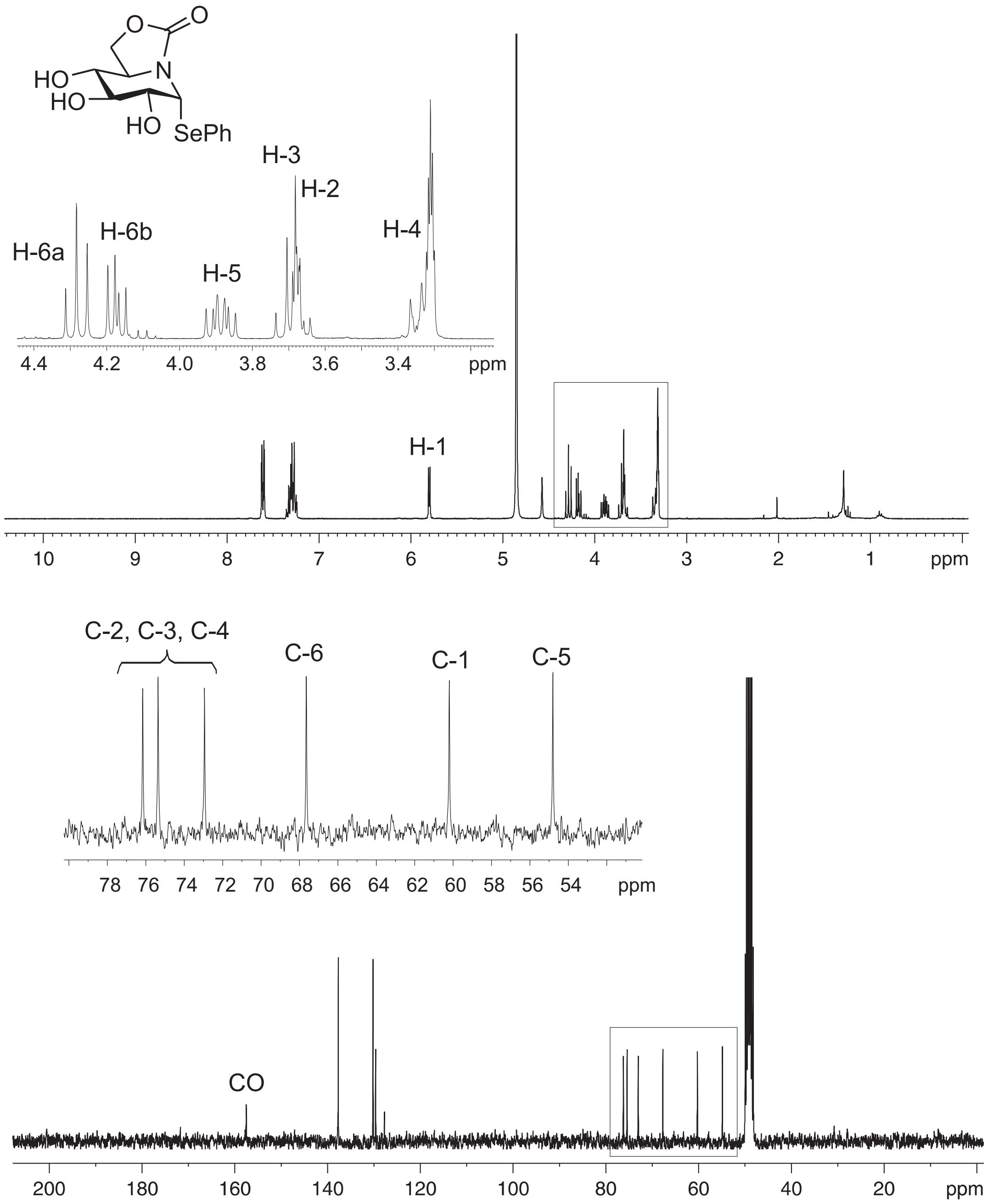


**Figure S8.** $^{1}H$ and $^{13}C$ NMR spectra (300 MHz and 75.5 MHz, $CD_3OD$) of **8**.

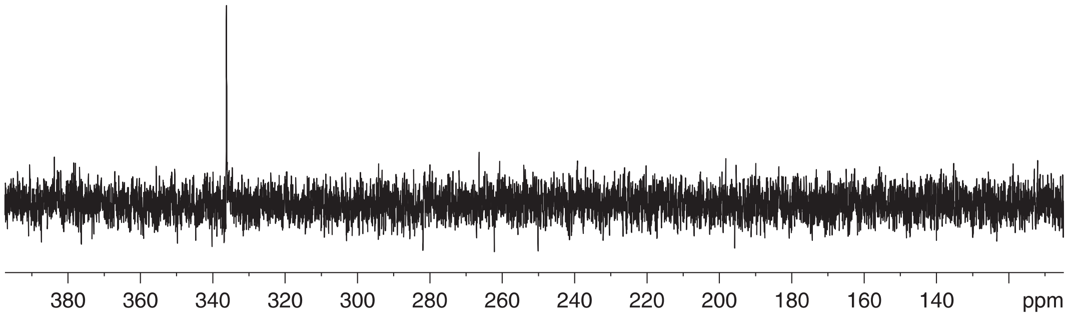


**Figure S9.** $^{77}Se$ NMR spectrum (95.4 MHz, $CD_3OD$) of **8**.

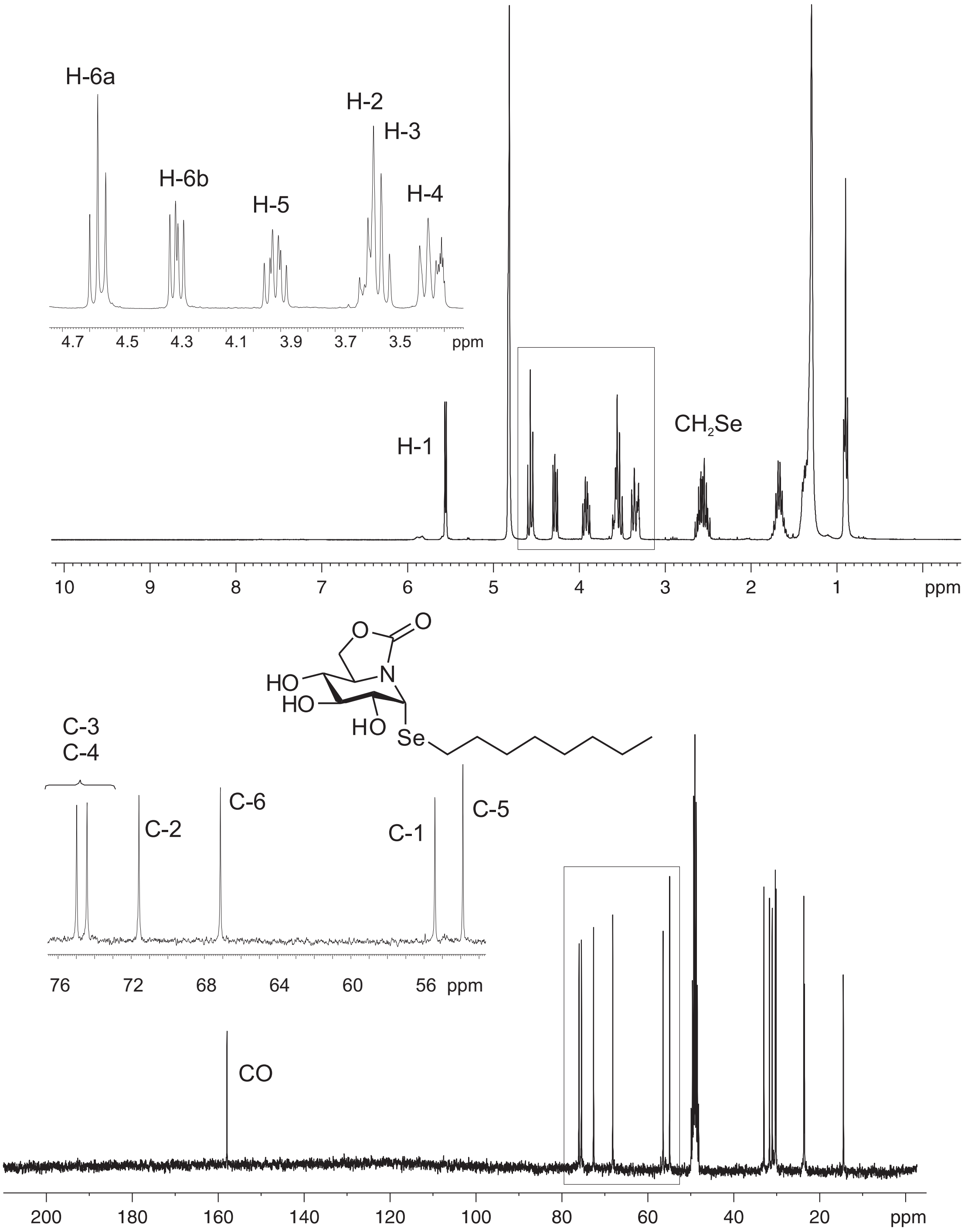


**Figure S10.** $^{1}H$ and $^{13}C$ NMR spectra (300 MHz and 75.5 MHz, $CD_3OD$) of **9**.

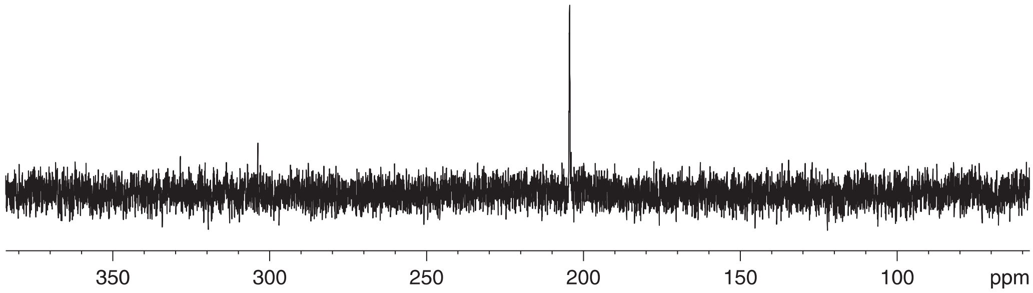


**Figure S11.** $^{77}Se$ NMR spectrum (95.4 MHz, $CD_3OD$) of **9**.

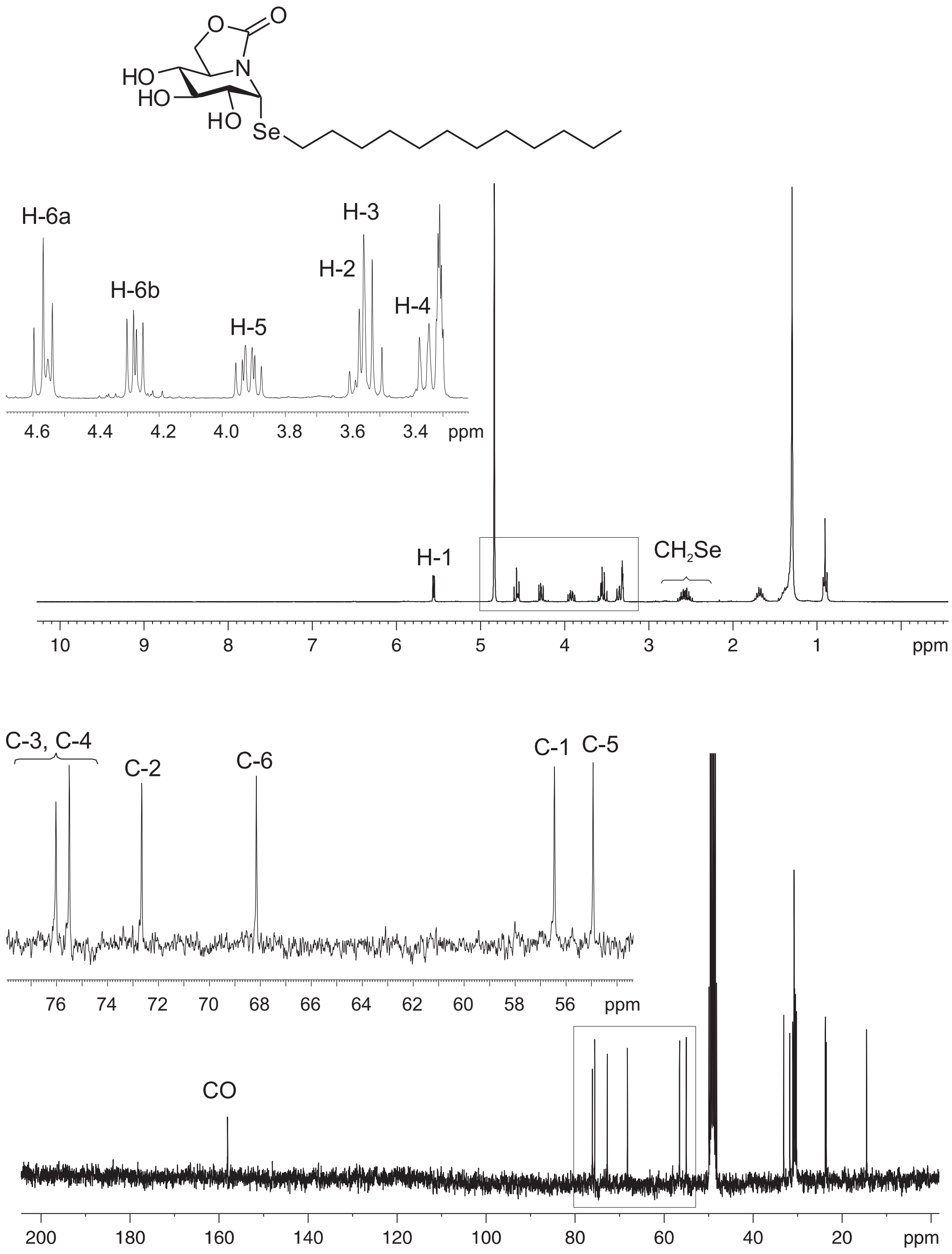


**Figure S12.** $^1$H and $^{13}$C NMR spectra (300 MHz and 75.5 MHz, $CD_3OD$) of **10**.

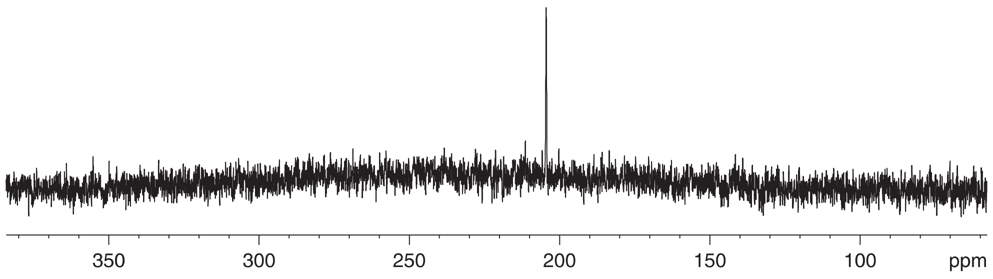


**Figure S13.** $^{77}$Se NMR spectrum (95.4 MHz, $CD_3OD$) of **10**.

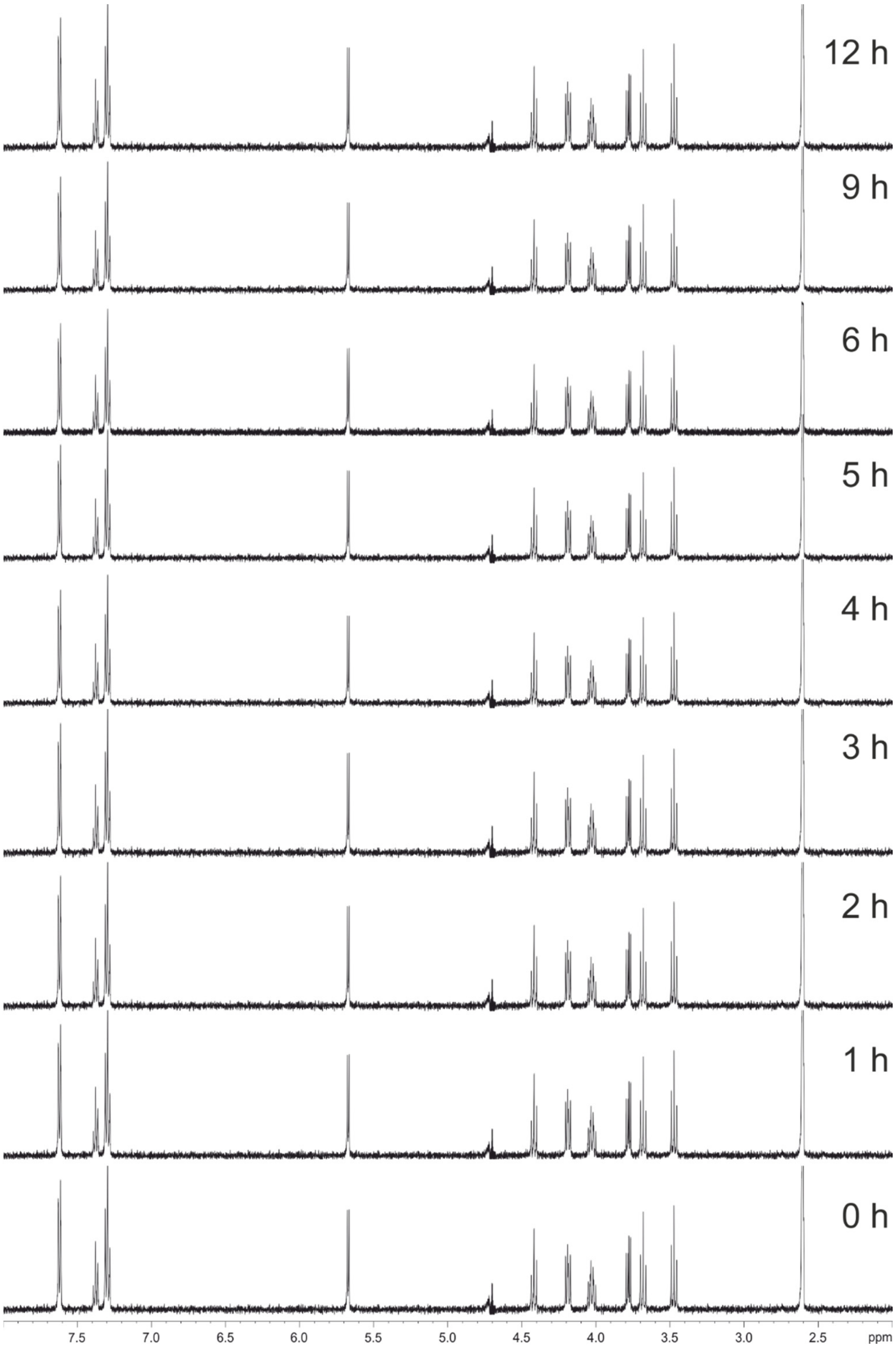


**Figure S14.** $^1$H-NMR-monitored kinetic evaluation of the stability of **8** (1 mM) in 9:1 $D_2O$-DMSO-$d_6$ at pH 4.4 (10 mM formate buffer).

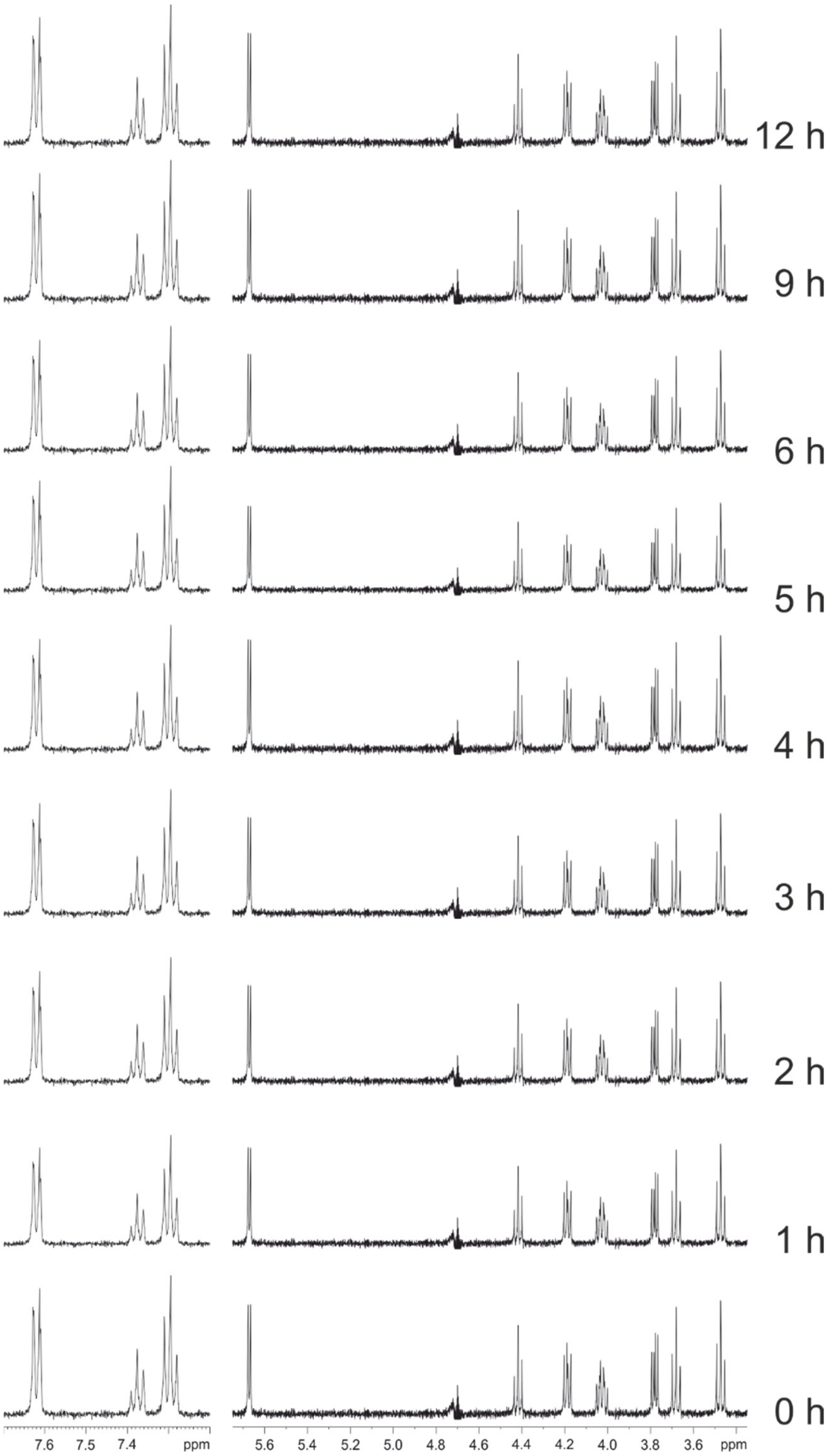


**Figure S15.** $^1$H-NMR-monitored (selected regions) kinetic evaluation of the stability of **8** (1 mM) in 9:1 $D_2O$-DMSO-*d*$_6$ at pH 4.4 (10 mM formate buffer).

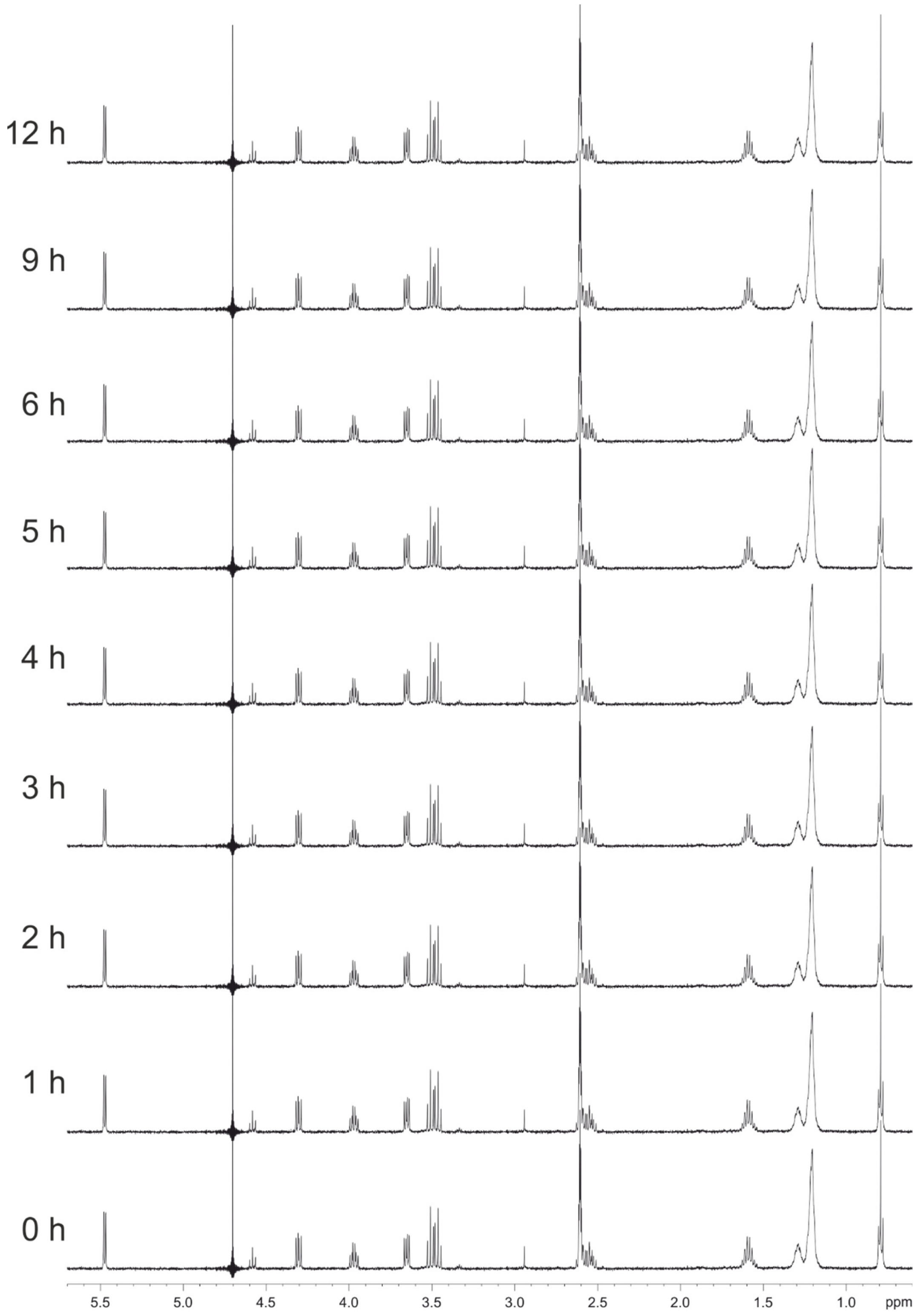


**Figure S16.** $^{1}$H-NMR-monitored kinetic evaluation of the stability of **9** (1 mM) in 9:1 $D_2O$-DMSO-$d_6$ at pH 4.6 (10 mM formate buffer).

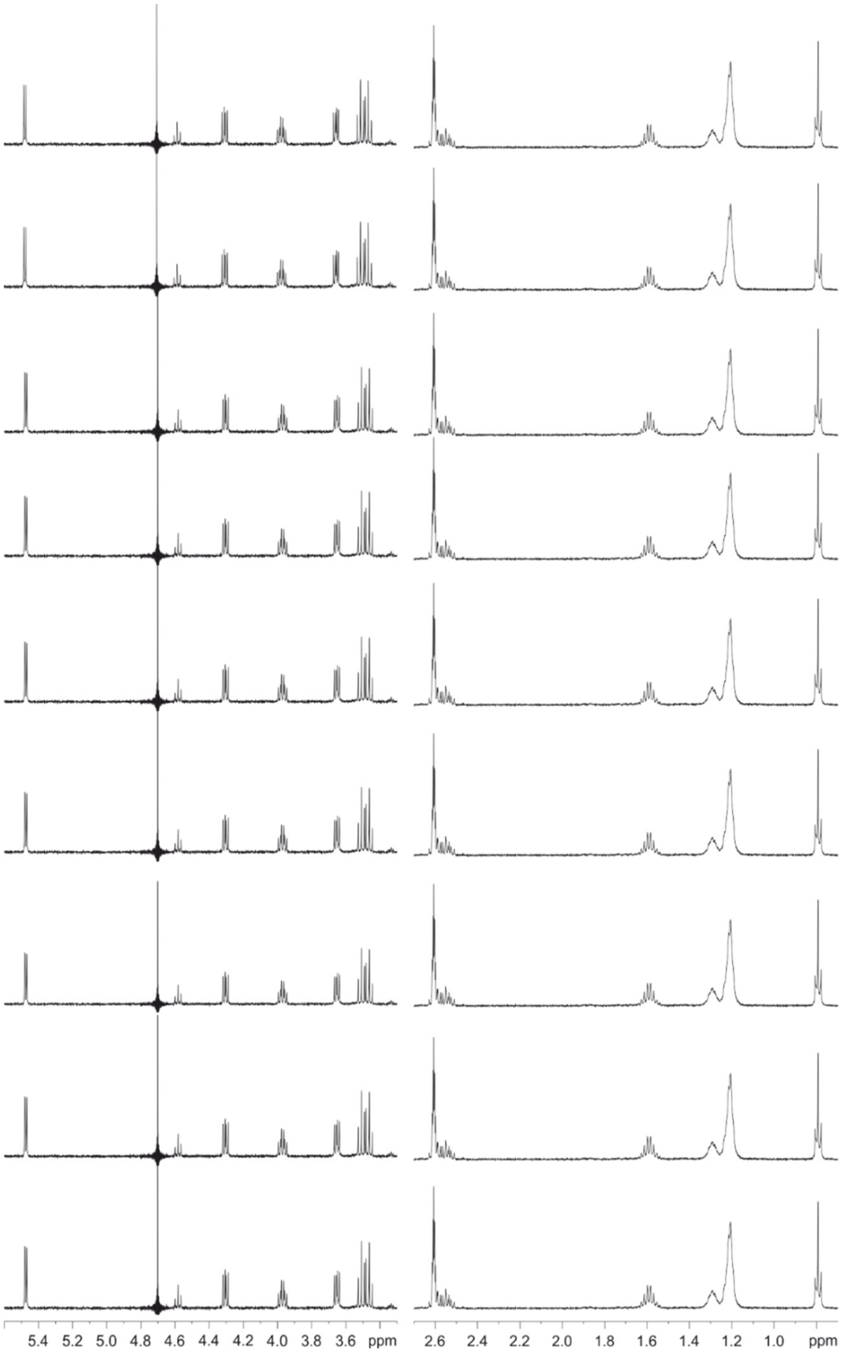


**Figure S17.** $^1$H-NMR-monitored (selected regions) kinetic evaluation of the stability of **9** (1 mM) in 9:1 $D_2O$-DMSO-$d_6$ at pH 4.6 (10 mM formate buffer).

**Table S1.** $GI_{50}$ values (µM) of the sp$^2$-IGLs (**8-14**) tested as antiproliferative agents by HTS.

| | **A549** | **SW1573** | **HBL-100** | **T-47D** | **HeLa** | **WiDr** |
|---|---|---|---|---|---|---|
| Compound | *Lung* | | *Breast* | | *Cervix* | *Colon* |
| **8** (Se–Ph) | >100 | >100 | >100 | >100 | >100 | >100 |
| **9** (Se–$C_8$) | 36( ± 9.5) | >100 | >100 | 71( ± 16) | 49( ± 14) | 64( ± 11) |
| **10** (Se–$C_{12}$) | 18( ± 4.3) | 28( ± 3.7) | 24( ± 8.1) | 21( ± 5.3) | 22( ± 5.5) | 22( ± 8.8) |
| **11** (NH–$C_8$) | >100 | >100 | >100 | >100 | >100 | >100 |
| **12** (NH–$C_{12}$) | 18( ± 4.4) | 30( ± 0.4) | 23( ± 7.0) | 23( ± 5.5) | 23( ± 7.7) | 22( ± 7.6) |
| **13** (S–$C_8$) | 58( ± 7.1) | >100 | 73( ± 38) | 95( ± 7.2) | 92( ± 12) | 89( ± 15) |
| **14** (S–$C_{12}$) | 16( ± 2.0) | 18( ± 1.2) | 17( ± 3.7) | 18( ± 1.9) | 17( ± 2.2) | 18( ± 0.2) |
